# Characterization of circular RNA profiles of oviduct reveal the potential mechanism in prolificacy trait of goat in the estrus cycle

**DOI:** 10.3389/fphys.2022.990691

**Published:** 2022-09-15

**Authors:** Zhipeng Sun, Qionghua Hong, Yufang Liu, Xiaoyun He, Ran Di, Xiangyu Wang, Chunhuan Ren, Zijun Zhang, Mingxing Chu

**Affiliations:** ^1^ Key Laboratory of Animal Genetics, Breeding and Reproduction of Ministry of Agriculture and Rural Affairs, Institute of Animal Science, Chinese Academy of Agricultural Sciences, Beijing, China; ^2^ College of Animal Science and Technology, Anhui Agricultural University, Hefei, China; ^3^ Yunnan Animal Science and Veterinary Institute, Kunming, China

**Keywords:** goat, oviduct tissues, prolificacy, estrus cycle, circular RNA, ceRNA network

## Abstract

The mammalian oviduct is functionally highly diverse during the estrus cycle. It provides a suitable milieu for oocyte maturation, sperm capacitation, fertilization, early embryo development and transportation. While there have been many studies of molecular mechanisms on the kidding number of goats, a systematic analysis by which the underlying circular RNAs (circRNAs) changes in the oviduct related to prolificacy traits is lacking. Herein, we present a comprehensive circRNA atlas of the oviduct among high- and low-fecundity goats in the follicular phase (FH vs. FL), luteal phase (LH vs. LL), and estrus cycle (FH vs. LH; FL vs. LL) to unravel their potential regulatory mechanisms in improving kidding number. We generated RNA sequencing data, and identified 4,078 circRNAs from twenty sampled Yunshang black goats. Many of these circRNAs are exon-derived and differentially expressed between each comparison group. Subsequently, eight differentially expressed (DE) circRNAs were validated by RT‒qPCR, which was consistent with the RNA-seq data. GO and KEGG enrichment analyses suggested that numerous host genes of DE circRNAs were involved in the hormone secretion, gamete production, fertilization, and embryo development processes. The competing endogenous RNA (ceRNA) interaction network analysis revealed that 2,673 circRNA–miRNA–mRNA axes (including 15 DE circRNAs, 14 miRNAs, and 1,699 mRNAs) were formed, and several target genes derived from the ceRNA network were associated with oviduct functions and reproduction, including *SMAD1*, *BMPR1B*, *IGF1*, *REV1*, and *BMP2K*. Furthermore, miR-15a-5p, miR-181b-5p, miR-23b-5p, miR-204-3p, and miR-145-5p might play important roles in reproduction. Finally, a novel circRNA, circIQCG, was identified as potentially involved in embryo development. Overall, our study provides a resource of circRNAs to understand the oviductal function and its connection to prolificacy trait of goats in the differentiation estrus cycle.

## Introduction

The kidding number is an important reproduction trait in goats and has a significant impact on the profitability of livestock animals. Understanding the reproductive physiology of a species is a necessary condition to improve animal yield (Sharma, 2000). For a long time, ovulation number and ovarian development have been considered the main factors affecting the number of lambs produced by sheep and goats (Notter, 2012). Therefore, the studies on fecundity in goats mainly focus on the ovaries, while the research on the molecular mechanism by which the oviduct regulates fertility is limited. Until now, some candidate genes have been found to regulate goat fertility, such as genes *BMP15* (Silva et al., 2005), *GDF9* and *KISS1* (Pramod et al., 2013; Lima et al., 2020), and *CDC25C* (Wang et al., 2021), are widely identified in the ovary, uterus, and other reproductive organs of goats. The oviduct, the channel connecting the ovary and uterus (Abe and Oikawa, 1992, 1993), is an active organ that provides a suitable environment for oocyte maturation, sperm capacitation, fertilization, and early embryo development and transportation (Hunter, 2003). It acts as a conduit for gamete during the periovulatory phase and nurtures and promotes the transportation of developing early embryos for implantation during the luteal phase (Hess et al., 2013). As noted above, these factors can affect the fecundity of goats. This highlights the necessity for further investigate the potential molecular mechanisms in the prolificacy trait of goat oviducts in different estrus cycles.

Several potential regulatory factors for regulating mammalian reproduction have been researched in recent years, including noncoding RNAs (ncRNAs) (e.g., long noncoding RNAs, micro RNAs) (Zhao et al., 2020; Ma et al., 2022). CircRNAs are a new type of regulatory ncRNA that have been rediscovered to regulate gene expression in eukaryote cells and are characterized by covalently closed-loop and splice connection sites formed by the covalent connection of the 3' and 5' ends (Memczak et al., 2013; Liang and Wilusz, 2014; Wilusz, 2018). Their special circular structure makes it more stable than mRNAs (Jeck and Sharpless, 2014), and they usually act as the sponges of microRNAs (miRNAs) (Zheng et al., 2016), interacting with the U1 snRNP to regulate gene expression (Li et al., 2015), which affects biological processes. In particular, it is worth mentioning that large amounts of circRNAs have been identified in various species (Salzman et al., 2013; You et al., 2015). Recently, RNA-seq of circRNA was performed in endometrium of dairy goats, during the estrus cycle showing that *Nipped-B-like* (*NIPBL*) and *calcium-responsive transcription factor* (*CARF*) may be involved in endometrial development by reducing the levels of their circRNA-transcriptional forms (Liu X. R. et al., 2021). However, their effects on the regulation of fecundity in the oviducts of animals have not been well elucidated. CircRNAs usually interact with RNA-binding proteins (RBPs) to perform their functions of translation, transcriptional regulation of target genes, and extracellular transport (Abdelmohsen et al., 2017; Zang et al., 2020). More recently, many studies have shown that circRNAs regulate gene expression in the biological processes and participate in the occurrence and development of various vital activities. For example, circRNA generated from circSF1 and the protein it encodes have been shown to extend the lifespan of flies (Baumann, 2020). CircUSP13 promotes goat myogenic cell differentiation and inhibits apoptosis by targeting *IGF1* through sponging miR-29c (Zhang et al., 2022c), whereas some other circRNAs are involved in the regulation of cashmere fineness (Zheng et al., 2020). Numerous studies have indicated that circRNA-derived specific miRNA regulates the reproductive process. Specifically, chi_circ_0008219 has been reported to regulate follicular development after sponging miRNA in ovarian follicles of preovulation of goats (Tao et al., 2017), and circ_026259 has been proven to promote the expression of *IGF1* by inhibiting oar-miR-29b in sheep Sertoli cells, which affected testicular development (Li et al., 2021). Meanwhile, in a study of the uterus, circ-8073 regulated *CEP55* by sponging miR-449a and promoting the proliferation of goat endometrial epithelial cells via the PI3K/AKT/mTOR pathway (Liu X. R. et al., 2018). Furthermore, in the endometrial epithelial cells (EECs) of dairy goats, the circRNA3175–miR182–TES (testin) pathway is a potential target of prereceptive endometrial development (Zhang L. et al., 2018). Previous reports have presented evidence that the development of preimplantation embryos is also regulated by circRNA (Dang et al., 2016). Nevertheless, whether circRNA expression in the oviduct can affect goat fecundity is not yet clear. To answer this question, we used the oviduct tissues of Yunshang black goats at the follicular and luteal phases to analyse the circRNA expression profiles and related ceRNA regulatory network, to explore the factors related to the kidding number.

Yunshang black goat (*Capra hircus*), is the first black mutton goat breed in China, with high reproductive performance, such as three kiddings per birth, and is an important genetic resource. It is an ideal model for studying high fecundity caused by its prolificacy performance. Here, to elucidate the molecular regulatory mechanisms of high fecundity in Yunshang black goats, we applied RNA-seq to systematically profile the oviduct transcriptomes of twenty goat individuals in different estrous cycles. A total of 4,078 circRNAs were identified from the high- and low-fecundity goat groups in the follicular and luteal phases. In the oviduct tissues, 174 circRNAs were differentially expressed between the FL and FH groups (97 were upregulated and 77 were downregulated), and 286 (167 were upregulated and 119 were downregulated), 558 (275 were upregulated and 283 were downregulated) and 467 (223 were upregulated and 244 were downregulated) DE circRNAs were identified in the LL vs. LH, LH vs. FH, and LL vs. FL comparison groups, respectively. Among these circRNAs, 4,020 were derived from 2,027 host genes. Furthermore, we explored the GO terms, and pathways of host genes connected to reproduction in the oviduct. Subsequently, we predicted the miRNAs that could be sponged by circRNAs, and circRNA−miRNA networks were constructed. A ceRNA network also constructed to elucidate the regulatory mechanisms of the prolificacy trait. Additionally, functional enrichment analyses of target genes further identified crucial correlated circRNA–miRNA–mRNA axes related to embryonic development and oviduct functions. We further performed an RT‒qPCR and observed that the expression level of miR-145-5p was opposite to the expression trends of circ_0001504 and *SMAD1*, while the expression trend of circ_0001504 and *SMAD1* were consistent, which might be associated with embryo development. Our findings will provide a better understanding of circRNAs in regulating prolificacy traits from the oviduct and may offer new insight into the kidding number of goats.

## Materials and methods

### Animals and ethics

Yunshang black goats, a new variety of domestic goats in China, were used in this study. Twenty healthy nonpregnant female goats (3 years old) were provided by the original breeding farm of Yixingheng Animal Husbandry Technology Co., Ltd. in Kunming city (Yunnan, China). The goats were free to eat and drink, with no significant differences in weight or body size. All animal procedures were approved by the Science Research Department (in charge of animal welfare issues) of the Institute Animal Sciences, Chinese Academy of Agricultural Sciences (IAS-CAAS), Beijing, China. Ethical approval was given by the Animal Ethics Committee of the IAS-CAAS (No. IAS 2021-23).

### Sample acquisition and tissue processing

**FIGURE 1:**
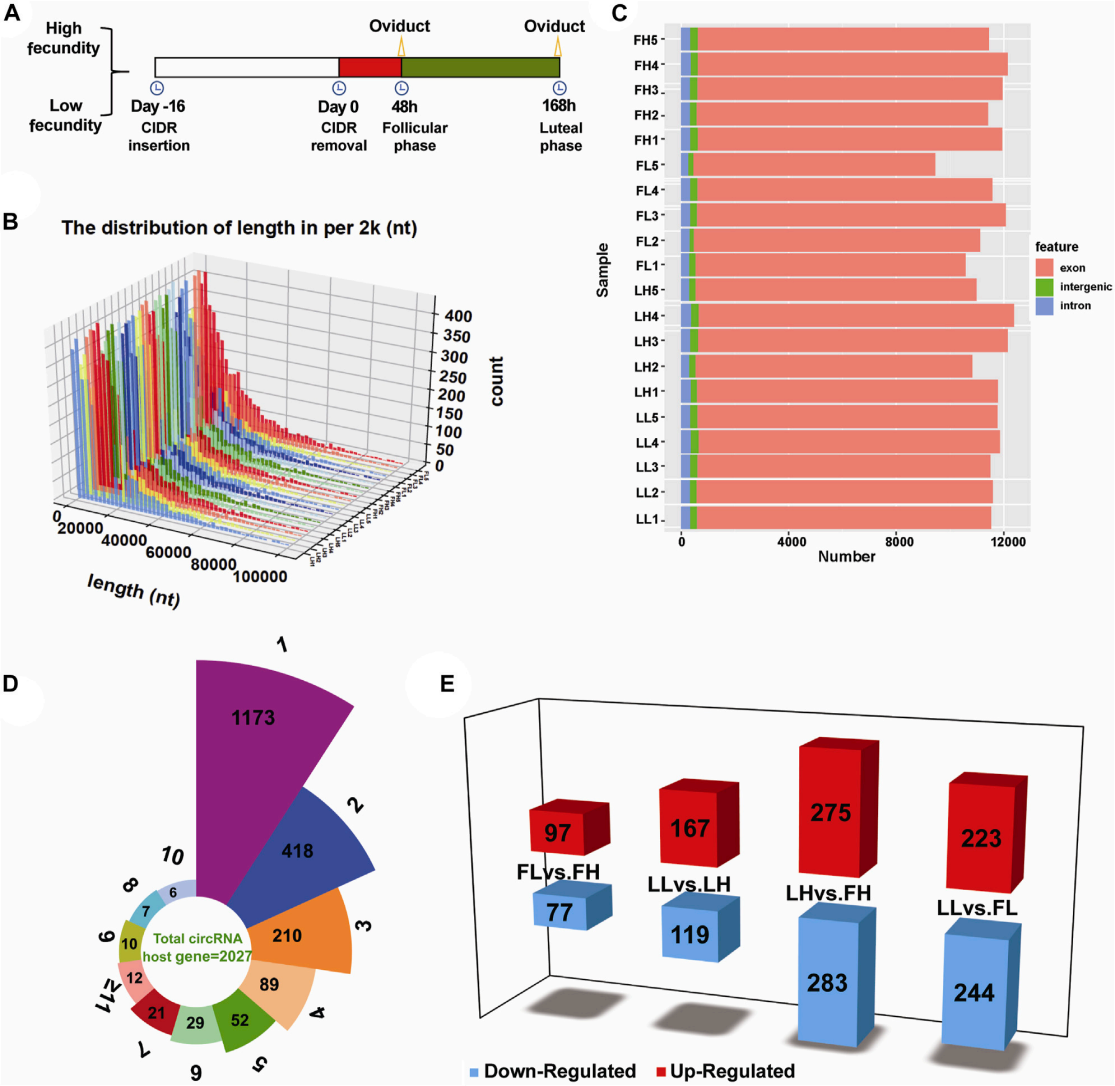
Genomic features of circRNAs in goat oviduct tissues. **(A)** Schematic representation of oviduct samples collected designed in this study. Twenty unique goats with high fecundity or low fecundity undergoing follicular phase oviduct samples (*n* = 10) or luteal phase oviduct samples (*n* = 10) were harvested for analysis. **(B)** The length distribution of circRNAs. **(C)** Function region of the detected circRNAs in terms of genomic origin. **(D)** The number of circRNAs produced from one gene (4,064 circRNAs from 2,027 host genes). **(E)** Statistics of DE circRNAs in the four comparisons; Red and blue represented upregulated and downregulated DE circRNAs, respectively.

Twenty oviduct tissues were collected at two timepoints covering the follicular and luteal phases from high-fecundity (*n* = 10, average kidding number 3.4 ± 0.42) and low-fecundity (*n* = 10, average kidding number 1.8 ± 0.27) Yunshang black goats and were divided into FH, FL, LH, and LF groups (*n* = 5 per group). In brief, all goats were treated with a controlled internal drug-releasing device (CIDR, progesterone 300 mg, Inter Ag Co., Ltd. New Zealand) to synchronize estrus. The CIDRs were removed after Day 16, and the time was set to 0 h. Next, five low-fecundity and five high-fecundity nanny goats were euthanized at 48 h (follicular phase; the average ovulation number of FH and FL groups was 5.4 ± 0.24 and 2.8 ± 0.37, respectively), and the remaining ten goats were euthanized at 168 h (luteal phase; the average corpus luteum number of LH and LL groups was 3.6 ± 0.40 and 1.6 ± 0.24, respectively) (Figure 1A). Oviduct samples were separated from reproductive organs after being slaughtered and were washed with ice-cold phosphate-buffered saline (PBS), quick-frozen in liquid nitrogen, and then stored at −80°C for RNA extraction.

### RNA library construction, sequencing, and data processing

The total RNA was isolated from each powdered oviduct tissue using TRIzol reagent (Invitrogen, Carlsbad, CA, United States) according to the manufacturer’s protocol. RNA samples were monitored for degradation and the extent of contamination by 1% agarose gel electrophoresis. The purity (OD 260/280 between 1.8 and 2.2) of the isolated RNA were quantified using a NanoPhotometer^®^ spectrophotometer (IMPLEN, CA, United States), and RNA integrity number (RIN) values were determined by an Agilent2100. An RIN of more than 8.0 for each sample was considered acceptable for RNA-seq. A Qubit^®^ 2.0 fluorometer (Life Technologies, CA, United States) with a Qubit^®^ RNA Assay Kit was used to measure the RNA concentration, and the integrity of the RNA was assessed using the RNA Nano 6000 Assay Kit of the Bioanalyzer 2100 System (Agilent Technologies, CA, United States).

The total RNA of per sample (3 mg, concentrations more than 300 ng/μL) was treated with the Ribo-Zero™ Eukaryote Kit (Epicentre, United States) to remove rRNA and before constructing the RNA-seq libraries. Sequencing libraries were prepared using the NEBNext^®^ Ultra^™^ Directional RNA Library Prep Kit for Illumina^®^ (NEB, United States) following the manufacturer’s instructions. Briefly, divalent cations in NEB Fragmentation Buffer were used for segmentation at high temperature. Random hexamers were used as primers to synthesize the first strand of complementary DNA (cDNA) in the M-MuLV reverse transcriptase system. Then, the RNA strand was degraded by RNaseH, and the second strand of cDNA was synthesized from dNTPs in the DNA polymerase I system, in which dTTP in the dNTPs was replaced by dUTP. The adenylated 3' ends of the DNA fragment prepared for hybridization were ligated to an NEB Next Adaptor with a hairpin loop structure. The library fragments were purified, and cDNA fragments with different lengths (250–300 bp) were screened by the AMPure XP system (Beckman Coulter, Beverly, United States). After that, PCR amplification was performed by adding 3 μL USER enzyme (NEB, United States) and screening the cDNA for aptamer ligation at 37°C for 15°min, followed by 95°C for 5 min (Liu et al., 2022). PCR was then performed with Phusion High–Fidelity DNA polymerase, Index (X), and primer universal PCR primers. Finally, the quality of the libraries were assessed using an Agilent Bioanalyzer 2100 system, and the products were purified using the AMPure XP system and subjected to high-throughput RNA sequencing (RNA-Seq) using the Illumina^®^ HiSeq 2,500.

The sequenced raw data were first cleaned to filter out the adaptor sequence reads. Second, the adapter-containing reads were removed, as were reads forming paired-end pairs with them when the content of N in the single-ended sequencing read exceeded 1% of the length that in the read. Finally, low-quality reads with more than 50% of the bases having a quality value ≤20 were deleted together with the reads constituting the paired-end pair. Then, the Q20, Q30, and GC contents of the clean data were calculated.

### Identification and quantification of oviduct circRNAs

High-quality reads were mapped to the reference genome using HISAT2 (v2.1.0) (Kim et al., 2015). The 20 nt anchors from the two ends for each unmapped read were religned to the reference genome using the Bowtie2 aligner, and subsequently submitted to Find_Circ software (v.1.1) (Memczak et al., 2013). CIRI2 software (v.2.0.5) (Gao et al., 2018) with default parameters was also used to identify circRNAs. Briefly, the PCC (paired chiastic clipping) signal, PEM (paired-end mapping) signal, and GT-AG shear site information were searched according to the BWA (Li and Durbin, 2009) comparison results, and junction reads were preliminarily determined. Then, based on the results of dynamic programming alignment and the read support number of circRNAs, the candidate circRNAs were compared with the genome annotation information. Briefly, the unmapped reads were processed to 20 nt anchors from both ends of the read. Finally, we combined the results of circRNAs identified by the two software programs and intersected the results according to the position of circRNAs on the chromosomes. To estimate estimating the relative expression of circRNAs, we calculated the total number of reads mapped across the circularized junctions and normalized the number of reads spanning the back-spliced junction to the total number of mapped reads (units in billionS) and read length using SRPBM (spliced reads per billion mapping) (Zheng et al., 2016). The R package DESeq2 was used to analyse the differential expression of circRNAs. circRNAs with *p* value ≤0.05 and fold change >1.2 or <0.83 were set as the threshold of differential expression.

### Bioinformatics analysis of differentially expressed circRNA host genes

The enrichment of the host genes of ED circRNAs was analysed by gene ontology (GO) and kyoto encyclopedia of genes and genomes (KEGG) to interpret the potential biological roles of the circRNAs. GO enrichment was based on the GO web resource (http://www.geneontology.org/) and KEGG annotation was performed using KOBAS 3.0 software (Mao et al., 2005).

### Protein‒protein Interaction analysis of host genes

The goat database was used as a reference. The PPI analysis of the host gene of DE circRNAs was carried out using the STRING database (organism: *Capra hircus*), the PPI analysis of the host gene of DE circRNAs was carried out. By extracting a series of host genes from the goat database, we constructed a PPI network with a score of >0.900 as the significant interaction.

### Validation of differentially expressed circRNAs

Eight coexpressed DE circRNAs were randomly screened from each group and quantified. Real-time quantitative polymerase chain reaction (RT‒qPCR) analyses were performed using QuantStudio^®^ 3 (ABI, Foster City, CA, United States). CircRNA primers were designed by Primer Premier six software, and GAPDH was used as an internal reference gene for circRNA and mRNA, and U6 was used as a referencr gene for miRNA. The primer information is listed in Table 1. All primers were synthesized by Sangon Biotech (Shanghai, China). Three replicates of each sample were analysed, and standard curves were established using a Roche Light Cycler^®^ 480 Ⅱ system (Roche Applied Science, Mannheim, Germany). RT‒qPCR was performed under the following conditions: 95°C for 5 min, then 40 cycles of amplification at 95°C for 5 s and annealing at 60°C for the 30 s (Liu et al., 2022). Relative quantification of circRNA expression was compared to the internal control and analysed using the 2^−ΔΔCt^ method dependent on the *t* test. The expression levels relative to the internal reference gene were computed by the 2^−ΔΔCt^ approach. Reverse transcription PCR (RT‒PCR) was carried out using 2 × Taq PCR Mix according to the manufacturer’s instructions. The PCR products of DE circRNAs were confirmed via 1.5% agarose gel electrophoresis, and the bands were extracted and subjected to Sanger sequencing.

**TABLE 1:** Information on RT-qPCR primers and amplification product sizes of DE circRNAs.

Gene name	Host gene	Primer sequence (5′–3′)	Product size (bp)
chi_circ_0006705	DGKB	F: AAA​GTG​GTG​GGA​AGC​AAG​GA	152
		R: AAG​GTC​CGC​AGT​CAC​ATT​CA	
chi_circ_0007400	RAP1B	F: TCC​ATC​ACA​GCA​CAG​TCC​AC	111
		R: TCA​CGC​ATG​GTG​CAA​ACT​TG	
chi_circ_0009933	RHOBTB3	F: CCG​CCC​TTA​CGA​GTC​ATT​G	146
		R: ATC​CCT​TCC​GTC​GTA​CTG​AC	
chi_circ_0011932	GKAP1	F: CAG​GGA​AAA​GAC​AAA​CCT​CTC​AC	148
		R: TGG​GGA​CAG​AAC​TAA​GTA​CTG​C	
chi_circ_0012627	REV3L	F: ATG​GTT​ATG​GAC​AGC​AGC​CAG​AAA​G	95
		R: AGT​GGA​AGA​TGG​ATT​GCC​TAA​AGC​C	
chi_circ_0022144	LRBA	F: TGG​GTA​GCC​ATG​GAC​AGG​AAC	147
		R: TGT​GCA​AGG​TAA​GAC​TGC​TGA​CT	
chi_circ_0023270	TEKT3	F: AAG​CCC​CTC​TTC​AGG​TAG​CC	136
		R: AGC​TCC​ATG​ATG​CTC​AAA​CAC​T	
chi_circ_0027916	FGFR2	F: GAA​AGC​GTG​GTC​CCG​TCT​GA	144
		R: CGC​AGC​CAC​GTA​AAC​TTC​TGG	
GAPDH	—	F: GCA​CCG​TCA​AGG​CTG​AGA​AC	138
		R: TGG​TGA​AGA​CGC​CAG​TGG​A	

### Network construction of circRNA-miRNA and competing endogenous RNA

Studies in recent years have shown that circRNA molecules are rich in miRNA binding sites, and act as miRNA sponges in cells, thus relieving the inhibition of miRNA on its target genes and increasing the expression level of target genes. miRanda (John et al., 2005) of miRNA target prediction software was used to predict the interactions between circRNAs and miRNAs in this study, and the circRNA-miRNA interaction network was constructed using Cytoscape software (v.3.9.0, Cytoscape Consortium, San Diego, CA, United States). Then, the gene-miRNA pairs were estimated using TargetScan (http://www.targetscan.org), and the ceRNA network of circRNA–miRNA–mRNA was visualized by Cytoscape v.3.9.0. In the ceRNA network, negatively coexpressed circRNA–miRNA or mRNA–miRNA pairs were selected by assessing a Pearson correlation coefficient < −0.4, and a *p* value of <0.05 as final ceRNA pairs. Functional analysis of the target genes, in the network, was mainly based on the GO and KEGG databases to further elucidate the regulatory functions of circRNAs.

### Statistical analysis

Statistical analyses were calculated by Student’s *t* test using SPSS 25.0, and GraphPad Prism v.9.3.1 was used to visualize the data. All data are expressed as the Means ± SEM. *p* values of <0.05 indicate statistical significance and are presented as *p < 0.05, **p < 0.01, and ***p < 0.001. We also calculated the expression levels of circRNAs, miRNA, and mRNA using RT‒qPCR.

## Results

### Transcriptome sequencing analysis of circRNAs in yunshang black goat oviducts

To characterize the role of circRNAs in goat prolificacy, we performed RNA-seq analyses of twenty oviduct samples in the follicular and luteal phases from high- and low-fecundity goats (Figure 1). The twenty oviduct tissues were divided into four groups: the follicular phase of high and low fecundity goats (FH:1–5; FL:1–5), and the luteal phase of high- and low-fecundity goats (LH:1–5; LL:1–5). Each sample was sequenced on an Illumina HiSeq 2500 sequencer yielding ∼100 million reads, which were mapped to the *Capra hircus* reference genome, and the average total mapping ratios of clean reads were 96.92%, 96.93%, 97.26%, and 96.69% in the FL, FH, LL, and LH groups (Supplementary Data Sheet S1). The percentages of GC content were 41.85%–51.45%, the Q30 was 91.90%–95.40% in the four groups, respectively (Supplementary Data Sheet S1), which represents the high quality of the sequencing data. In total, there were 4,078 distinct circRNA candidates from all tissues (Supplementary Data Sheet S1), and the length distribution of most circRNAs differed, with most lengths primarily in the range from 100 to 20,000 nucleotides (Figure 1B). We further analysed the compositions of the significantly expressed circRNAs in terms of genome origin. They were mainly derived from exons, intergenic regions, and introns, most of which were identified in the exon regions, followed by introns (Figure 1C). In addition, a Circos plot displayed the distribution and expression of detected and significantly expressed circRNAs on *Capra hircus* of the top ten chromosomes, and the chromosomes 1, 2, and 3 had the highest proportion, as shown in Supplementary Figure S1. Notably, these circRNAs were present on any chromosome commonly stained with No. 1 to 29 autosomes and X chromosomes, indicating the diversity of circRNA functions.

### Differential expression analysis of circRNAs

In total, 4,078 distinct circRNA candidates were identified and the expression levels per group were normalized to SRPBM, as presented in Supplementary Figure S2. The SRPBM interval of the majority of circRNAs ranged from 500 to 2,000 (Supplementary Data Sheet S1), with percentages exceeding 35%. Of these, 4,020 were derived from 2,027 host genes, indicating that one gene can produce one or multiple circRNAs. As shown in Figure 1D, more than 50% of these host genes (1173) were generated to one circRNA, 418 host genes produced two circRNAs, and only 12 host genes produced more than 11 circRNAs. These circRNAs were named after the host genes (Supplementary Data Sheet S1). For example, *mitogen-activated protein kinase 1* (*MAPK1*) produces two subtypes of circRNAs (chi_circ_0030657 and chi_circ_0030657). The expression analysis of these host genes of circRNA transcripts found that various circRNAs may play key roles in regulating reproduction and embryonic development, such as *PIK3CA* (host gene of circPIK3CA), *SMAD1* (host gene of circSMAD1), *ESR1* (host gene of circESR1), *DNMT3A* (host gene of circDNMT3A), and *IGFBP5* (host gene of circIGFBP5).

Subsequently, DE circRNAs were identified according to the normalized expression with fold change >1.2 or <0.83 and *p* < 0.05. Of the four comparison groups, that is, FL vs. FH, LL vs. LH, LH vs. FH, and LL vs. FL. The LH vs. FH group exhibited the largest number of DE circRNAs (558, including 275 upregulated and 283 downregulated), as show in Figure 1E. There were 174 (97 upregulated and 77 downregulated) DE circRNAs identified in FL vs. FH, 286 DE circRNAs (167 upregulated and 119 downregulated) characterized in the LL vs. LH, and 467 DE circRNAs (223 upregulated and 244 downregulated) identified in LL vs. FL (Figure 1E). An interesting finding that is the process of oviduct transition from follicular phase to luteal phase showed more host genes as well as more types of DE circRNAs transcripts (558), reaching the highest levels in the high fecundity groups (Figure 1E; Supplementary Data Sheet S1). This indicates that more genes and circRNAs played roles in the transformation of the oviduct from the follicular phase to the luteal phase, and these altered genes and circRNAs might be the reason for the difference of fecundity in goats. In particular, we found that were 97 overlapping DE circRNAs were identified in both LH vs. FH and LL vs. FL groups; of the 14 DE circRNAs also coexpressed upon comparing the FL vs. FH and LL vs. LH groups; there were no DE circRNAs coregulated in the four comparison groups (Supplementary Figure S3). In addition, K-means and SOM cluster analyses were used to cluster the genes of the four groups, and the potential relationship between them was observed more intuitively (Supplementary Figure S4).

### Validation of circRNAs by sanger sequencing and RT‒qPCR

**FIGURE 2:**
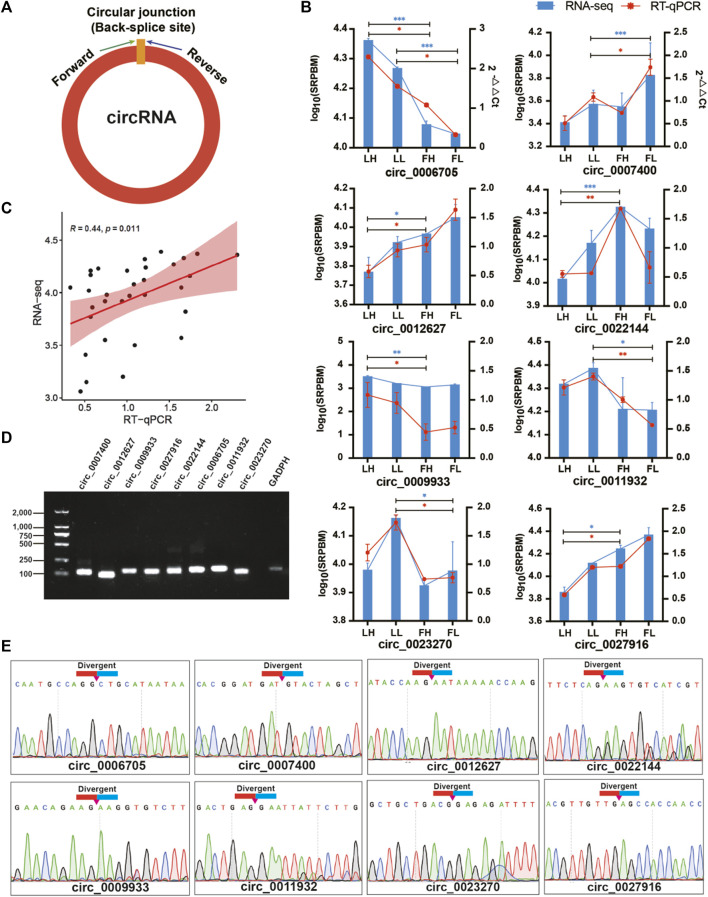
Verification of eight DE circRNAs. **(A)** Divergent primers were designed for circRNA validation. **(B)** Relative expression of circRNAs in goat oviducts were determined by RT‒qPCR and compared with the results of DE circRNAs in RNA-seq of the four groups. Data are the means ± SEM of three experiments of five biological repeats. *p < 0.05, **p < 0.01, ***p < 0.001. **(C)** Scatter plots show the correlation of circRNA expression between RNA-Seq and RT-qPCR. Pearson correlation coefficient (R) and the *p* value is shown in the top left corner. **(D)** RT‒PCR products of DE circRNAs were electrophoresed on a 1.5% agarose gel. **(E)** Sanger sequencing-based validation of the circular junctions of circRNA.

To ensure the accuracy of the RNA-seq strategy, eight DE circRNAs were randomly selected, and specific qPCR primers were designed within the junction regions of the circRNAs (Figure 2A). The expression levels of DE circRNAs indicated that the differences observed in our RNA-seq results were well validated well when assessed by RT‒qPCR assessment on independent samples (Figure 2B). These results collectively suggested the reliability of the DE circRNAs identified using RNA-seq. Additionally, the log_10_(SRPBM) values obtained based on the RNA-seq data showed a significant positive correlation with the 2^−ΔΔCt^ values obtained from RT‒qPCR (Pearson correlation coefficient, *R* = 0.44, *p*-value < 0.05; Figure 2C). Electrophoresis in a 1.5% agarose gel electrophoresis showed the expected sizes of the individual bands for each selected DE circRNA (Figure 2D). Sanger sequencing was then performed on the generated fragments, and the results showed that the sequence information of the circular junction of these circRNAs were the same as that of circRNAs sequencing, which proved that circRNAs were looped (Figure 2E).

### Functional enrichment for host genes of differentially expressed circRNAs

**FIGURE 3:**
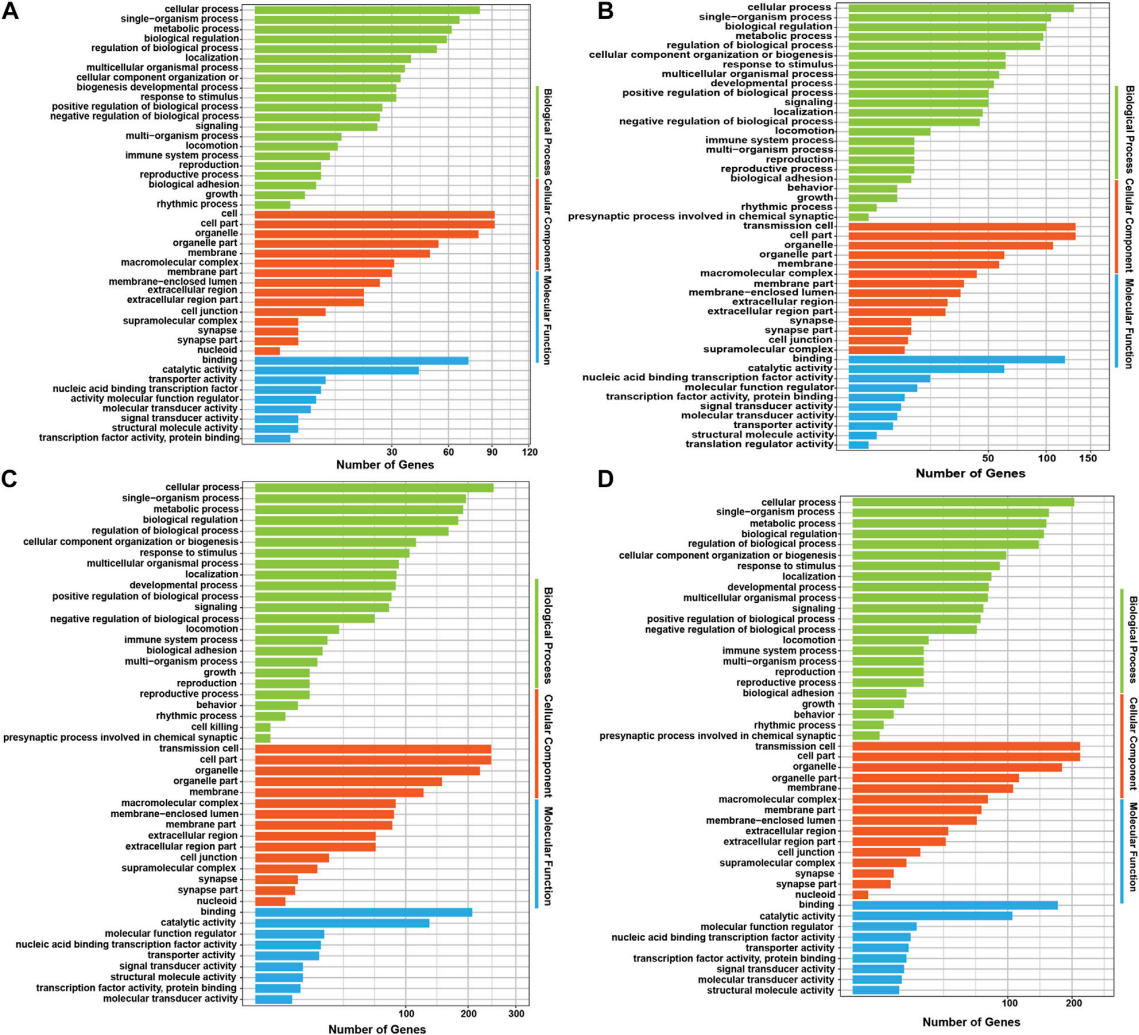
GO analysis of the host genes with differentially expressed circRNAs (DE circRNAs). The GO annotation analysis results of **(A)** FL vs. FH, **(B)** LL vs. LH, **(C)** LH vs. FH, and **(D)** LL vs. FL groups.

Analysing the functions of host genes can better reflect the roles of circRNAs. Based on the DE circRNA–derived genes, we used online tools for GO analysis and KOBAS 3.0 for KEGG enrichment analysis to predict their potential functions in gamete maturation, fertilization and embryonic development, and the results are shown in Supplementary Data Sheet S3, respectively. The GO annotation analysis of host genes from each group revealed that circRNAs might be involved in multiple processes that are crucial for reproduction and oviduct function. There were 447, 797, 754, and 607 terms significantly enriched (*p* value <0.05) in the FL vs. FH, LL vs. LH, LH vs. FH, and LL vs. FL comparison groups, respectively (Supplementary Data Sheet S3). Among the four comparison groups, the top ten terms of biological process (BP), cell components (CC), and molecular functions (MF) are shown in the circular diagrams in Figure 3 (Supplementary Data Sheet S3). Interestingly, in the comparison groups of LL vs. LH, LH vs. FH, and LL vs. FL, the top GO terms all contained cell part (GO: 0044464), intracellular (GO: 0005622), and binding (GO: 0005488), *etc*., However, the FL vs. FH comparison group differed from the other three comparison groups, which the most enriched items were protein metabolic process (GO: 0005623) and cellular protein metabolic process (GO: 0005622). In addition, we also found that GO terms related to gamete activity, such as gamete generation (GO: 0071902), positive regulation of oogenesis (GO: 1905881), flagellated sperm motility (GO: 0043266), and positive regulation of oocyte development (GO: 0070862) were mainly annotated in the follicular phase (LL vs. LH). Moreover, cell migration (GO: 0016477) was specifically enriched in the luteal phase of the oviduct (LL vs. LH). Nevertheless, the terms of developmental process (GO: 0032502), regulation of cell development (GO: 0060284), growth hormone secretion (GO: 0030252), embryonic placenta development (GO: 0001892), thyroid hormone receptor binding (GO: 0046966), the developmental process involved in reproduction (GO: 0003006), and reproductive process (GO: 0022414) were associated with cell development, transport, and hormone secretion, of which were annotated in the regulatory mechanism of the follicular to luteal transition (LH vs. FH and LL vs. FL). The host genes *BMPK8*, *STK39*, *MAP4*, *AP5M1*, *PALMD*, *GTF3C3*, *CDK13*, *EV15*, *MAPK3*, *NF1*, and *PIK3CA* of the DE circRNAs that are involved in the regulation of cell proliferation, migration, apoptosis, embryonic development, and reproduction associated with the oviduct. These results suggest that the oviduct may play a key role in the estrus cycles, thus affecting the kidding numbers of goats.

**FIGURE 4:**
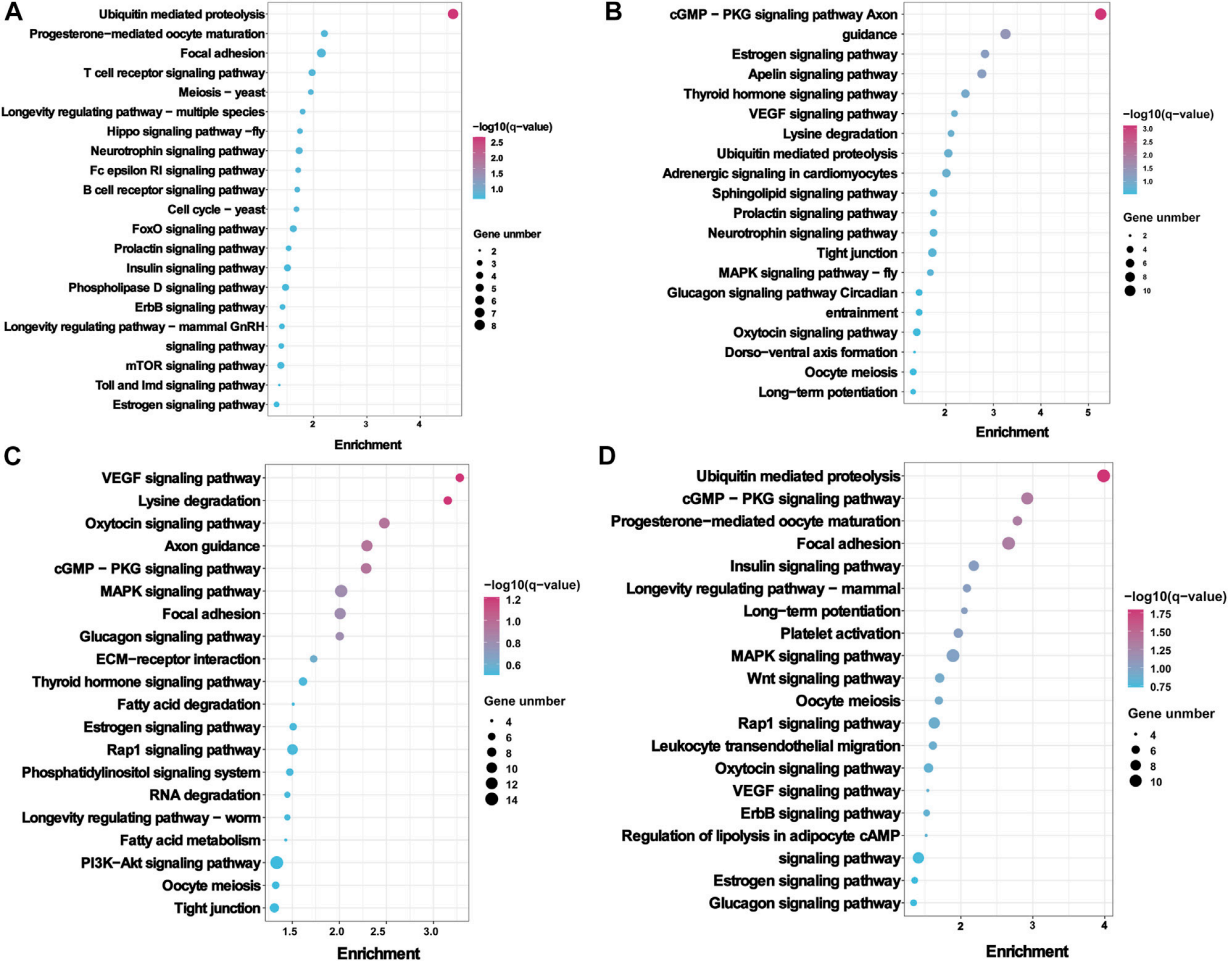
KEGG enrichment analysis of the host genes with differentially expressed circRNAs (DE circRNAs). The KEGG enrichment analysis results of **(A)** FL vs. FH, **(B)** LL vs. LH, **(C)** LH vs. FH, and **(D)** LL vs. FL groups. The top 20 KEGG pathways of each comparison group were visualized.

Worthy to note, the KEGG pathway showed that the host genes of DE circRNA were most enriched in the reproductive signalling pathways, including progesterone–mediated oocyte maturation (ko04914), insulin signalling pathway (ko04910), prolactin signalling pathway (ko04917), estrogen signalling pathway (ko04915), oocyte meiosis (ko04114), thyroid hormone signalling pathway (ko04919), and glucagon signalling pathway (ko04922). in the FL vs. FH, LL vs. LH, LH vs. FH, and LL vs. FL groups (Figure 4, Supplementary Data Sheet S3). Notably, the above-enriched pathways were mainly related to *ITPR1*, *MAPK1*, *ADCY9*, *CUL1*, *ADCY9*, *ITPR1*, *PRKCE*, *MYH11*, and *GAM2* and were involved in regulating embryo development and oviduct function, indicating the potential role of these DE circRNAs in Yunshang black goat prolificacy.

### PPI network analysis of host genes

**FIGURE 5:**
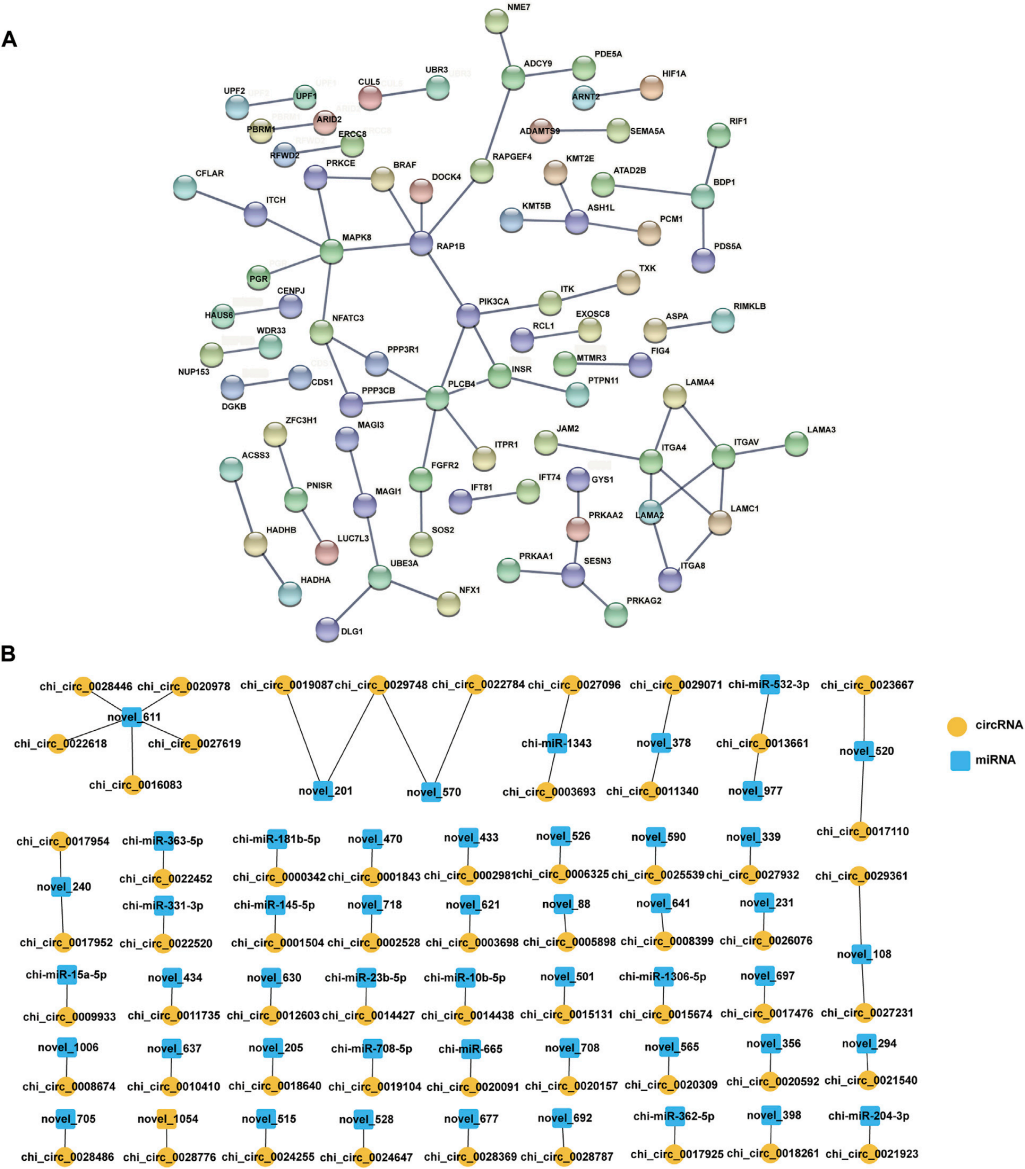
Interaction network analysis. **(A)** PPI network analysis for host genes of DE circRNAs, a score of >0.900. **(B)** The predicted targeting pairs of circRNA and miRNA. Orange circles represent circRNAs, the blue square represents miRNAs, *p*-value < 0.05, Pearson correlation coefficient < −0.4.

Furthermore, the host genes of all DE circRNAs in the four comparison groups were analysed using the STRING database, and the PPI network of host genes (Figure 5A) was constructed, which contained 68 protein‒protein pairs. Notably, analysing the protein nodes in the network, it was found that RAP1B had five nodes, including RAPGEF4, DOCK4, BRAF, PIK3CA, and MAPK8. Accumulating data indicate that RAP1B is involved in cell proliferation, development, differentiation, and adhesion (Chen et al., 2008; Liu M. et al., 2018). PIK3CA and MAPK8 are associated with female fertility and cell development, respectively (Chang et al., 2018).

### Analysis of the targeting relationships of circRNA-miRNA pairs

The binding sites of 4,078 circRNAs combined with miRNAs were predicted by miRanda software to further identify the key roles of circRNAs that affect the fertility traits of goats. A total of 2,657 circRNA–miRNA pairs were confirmed (Supplementary Data Sheet S4), and 61 circRNA-miRNA pairs (Figure 5B, Supplementary Data Sheet S4) were ultimately determined according to *p* value <0.05, and a Pearson correlation coefficient < −0.4, which covered 14 known miRNAs, 36 novel miRNAs and 59 circRNAs (Supplementary Data Sheet S4). And then, we found that 5 DE circRNAs were bound with five miRNAs in the LL vs. LH comparison group, 11 DE circRNAs were bound with 10 miRNAs in the LH vs. FH comparison group, and 8 DE circRNAs were bound with eight miRNAs in the LL vs. FL comparison group (Supplementary Data Sheet S4).

### CeRNA network construction and functional annotation of target genes

**FIGURE 6:**
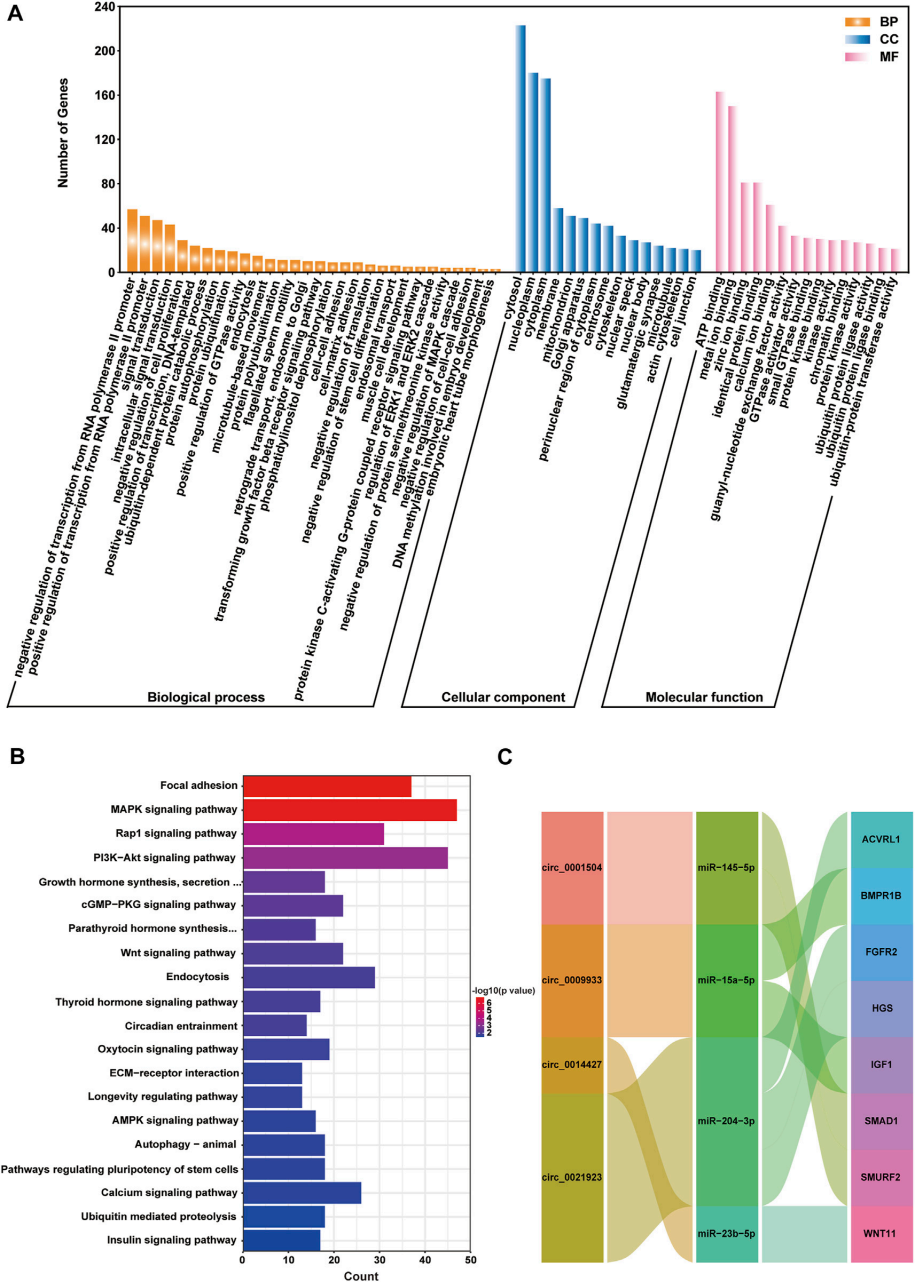
The circRNA–miRNA–mRNA (ceRNA) interaction network, and function analysis of target genes. **(A)** GO classification and **(B)** KEGG enrichment analysis of the target genes. **(C)** Sankey plot of the circRNA-miRNA-mRNA regulatory network enriched for reproduction-related genes.

Recently, circRNAs have been proven to be rich in miRNA binding sites and to act as miRNA sponges in cells, thereby releasing the inhibitory effect of miRNAs on their target genes and increasing their expression levels (Liu et al., 2019). This regulatory mechanism is referred to as ceRNA. To further investigate the regulatory role of circRNAs on the biological function of the oviduct, circRNA–miRNA–mRNA regulatory networks based on circRNA-miRNA pairs and the target genes of miRNAs were constructed as a ceRNA network. After removing novel miRNAs and novel mRNAs, 2,673 circRNA–miRNA–mRNA axes were formed, including 1,699 mRNAs (Supplementary Figure S5, Supplementary Data Sheet S4). Notably, GO annotation analysis revealed that mRNAs were derived from genes in the ceRNA network; in BP, and were mainly involved in reproduction, such as the negative regulation of cell proliferation, flagellated sperm motility, microtubule-based movement, transforming growth factor-beta receptor signalling pathway, DNA methylation involved in embryo development, biological adhesion, and protein modification (Figure 6A; Supplement Data Sheet S4); in CC, they mainly included cytosol, nucleoplasm, and cell junction (Figure 6A); in MF, they mainly clustered with binding (e.g., androgen receptor binding), enzymatic activity, and transcription factor activity (Figure 6A). Subsequently, KEGG enrichment showed that mRNAs were most involved in the reproductive signalling pathways, such as MAPK, PI3K-Akt, cGMP-PKG, Wnt, thyroid hormone, oxytocin, insulin, ECM-receptor interaction, and growth hormone synthesis, secretion, and action, and so on (Figure 6B; Supplement Data Sheet S4). These GO annotation and KEGG enrichment results suggest that DE circRNAs may have key roles in regulating fecundity in Yunshang black goats by participating in embryonic development, gamete viability, and cell proliferation.

In the ceRNA network, no genes were regulated by circRNA–miRNA pairs in the FL vs. FH group; 105 genes were regulated by circ_0009933–miR-15a-5p pairs in the LL vs. LH group; 811 genes were regulated by six circRNA–miRNA pairs (circ_0001504–miR-145-5p, circ_0000342–miR-181b-5p, circ_0009933–miR-15a-5p, circ_0014427–miR-23b-5p, circ_0022452–miR-363-5p, circ_0021923–miR-204-3p) in the LH vs. FH group; and 296 genes were potentially regulated by circ_0022452–miR-363-5p and circ_0001504–miR-145-5p pairs in the LL vs. FL group. Interestingly, there were more circRNA–miRNA–mRNA regulatory relationships during the transition from the follicular phase to the luteal phase in the high-fecundity group (LH vs. FH), and in the ceRNA network, the chi_circ_0000342-chi-miR-181b-5p axis targets 38 genes, including the embryonic development-associated gene *YAP1*; the chi_circ_0009933-chi-miR-15a-5p axis was identified, which targets 122 genes, including the reproductive-associated genes *BMPR1B* and *IGF1*; chi-miR-23b-5p regulates *WNT11* expression and is sponged by chi_circ_0014427; and miR-204-3p was predicted to target chi_circ_0021923 and regulate *ACVRL1*, *FGFR1* and *HGS*. In addition, the target genes *SMAD1*, *SMURF2*, *ACVRL1*, *HGS*, *BMPR1B*, and *IGF1* were involved in the transforming growth factor-beta receptor signalling pathway or signalling pathways regulating the pluripotency of stem cells. Furthermore, target genes related to embryonic development were selected, and a circRNA–miRNA–mRNA regulatory network was constructed, which consisted of 4 DE circRNAs, four miRNAs, and eight mRNAs (Figure 6C).

### CircIQCG serves as a miRNA sponge of miR-145-5p to regulate SMAD1 expression

**FIGURE 7:**
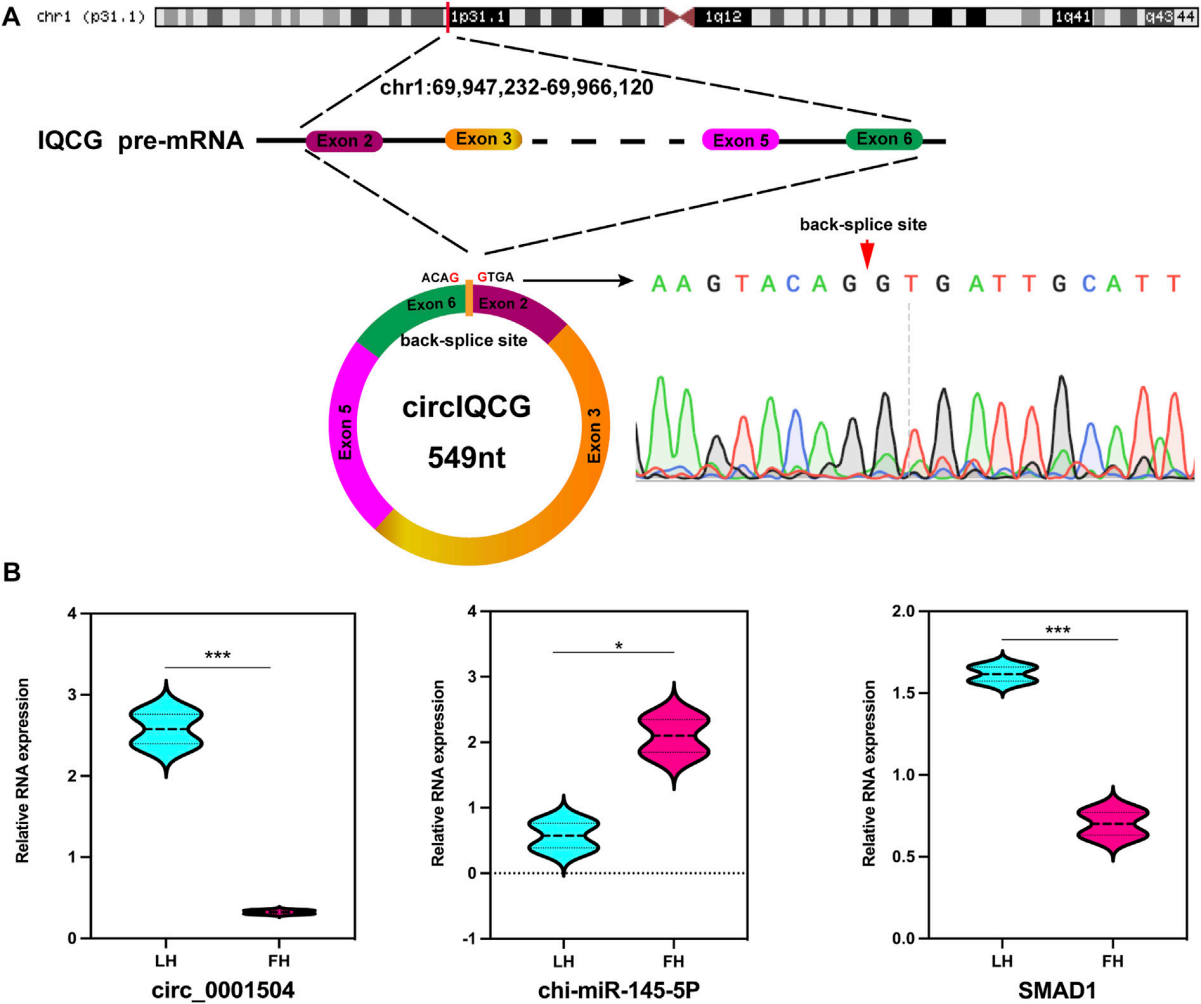
Characterization and expression of circIQCG. **(A)** The genome size and sequence reported in the circBase database were determined, and the head-to-tail splicing site of circIQCG in that RT-PCR product was confirmed by Sanger sequencing. **(B)** RT-qPCR analysis for the relative RNA expression.

We focused on the DE circRNAs during the transition from the follicular phase to the luteal phase in high-fecundity goat oviducts; of these, an upregulated circRNA in the luteal phase, chi_circ_0001504, was predicted to be adsorbed miR-145-5p. Therefore, we investigated the biofunction of chi_circ_0001504 in the oviduct, which originated from the *IQCG* gene of chromosome 1 (chr1:69,947,232–69,966,120), and finally consisted of head-to-tail splicing of exons 2 and 6 (Figure 7A), named circIQCG. Stability has long been considered one of the most important features of circRNA (Vo et al., 2019). Consequently, we confirmed the circIQCG head-to-tail splicing by Sanger sequencing and validated it using circBase data (Figure 7A). As a potential target gene of miR-145-5p, *SMAD1* might be regulated by the circIQCG–miR-145-5p axis, which is a known regulator of the TGF-β signalling pathway and affects embryonic development. Subsequently, RT‒qPCR results indicated that *SMAD1* and circIQCG were the same expression trends (Figure 7B), while circIQCG/*SMAD1* and miR-145-5p showed opposite expression trends (Figure 7B). The primers information is listed in Supplementary Data Sheet S5. These results preliminarily confirmed the direct interaction between circIQCG/*SMAD1* and miR-145-5P.

## Discussion

Reproduction is a complex process in which transcriptional regulation is one of the important factors affecting reproductive traits (Luo et al., 2019). The improvement in mammalian fecundity might be enhanced by the identification and development of various regulatory factors, including circRNAs, using molecular breeding techniques. As the most abundant noncoding RNAs in the eukaryotic transcriptome, circRNAs are important regulators of gene expression at the transcriptional, posttranscriptional, and translational levels and miRNA function (Patop et al., 2019). Numerous studies have demonstrated the role of circRNAs in regulating mammalian fecundity (Zou et al., 2020; Liu Y. F. et al., 2021; Liu et al., 2022). Tao et al. detected 13,950 circRNAs in goat preovulatory follicles, of which 37 circRNAs were differentially expressed (Tao et al., 2017). Liu et al. analysed 2,331 DE circRNAs in goat uterine membranes (Liu X. R. et al., 2021). Similarly, previous studies have analysed circRNAs in goat ovaries (Liu et al., 2022) and pituitary glands (Liu Y. F. et al., 2021), and some DE circRNA as miRNA sponges were identified, which have the potential to regulate fertility in goats. Although circRNAs have been identified in different reproductive organs of goats, however, their biological role in the oviduct remains unclear. Herein, we identified and characterized the expression patterns of circRNAs in the oviduct in the follicular and luteal phases by RNA-seq as well as bioinformatics analysis in goats. Further analysis showed that the circRNAs identified were mainly generated by cyclization of gene exons (Zhang M. L. et al., 2018), in the sheep (Lv et al., 2020) and cattle (Liu et al., 2020), indicating similar origins of circRNAs in different species. In our present study, we found that the majority of circRNAs are less than 10,000 nt in length, which is consistent with the results of a previous study in goat ovary (Liu et al., 2022), but longer than circRNAs identified in goat endometrial (Li et al., 2022), indicating that the longer the sequence, the greater the corresponding increase in the difficulty of circRNA loop formation. Moreover, most of the identified circRNAs were typically distributed on chromosomes 1-11, and the expression of circRNAs in the luteal phase was more abundant than that in the follicular phase of the oviduct, indicating that the functions of circRNAs in the oviduct are diverse.

The function of circRNAs in the tissues of animals is correlated with host genes. Therefore, the functions of the DE circRNAs identified in this study were mainly predicted analysing host genes GO annotation and KEGG enrichment. Most of the GO terms and KEGG pathways related to cell development, migration, hormone secretion, and reproductive processes were enriched. Notably, the host genes *PLCB4*, *FKBP5*, and *ESR1* of chi_circ_0017802, chi_circ_0026680, and chi_circ_0013463 were enriched in the estrogen-related signalling pathways. Previous study has shown that estrogenic changes during the different oestrus stages caused the oviductal epithelial cells (OECs) to change from ciliated cells in the follicular phase to secretory cells in the luteal phase, which are important for embryonic development and transportation (Abe, 1996; Lyons et al., 2006). It is noteworthy that abundant biological adhesions were enriched in the follicular and luteal phases of the oviduct. As the most important location of origin of life activity, the oviduct play an important role on gamete viability, fertilization, and early embryo transport, which critically depend on adhesion (Budna-Tukan et al., 2019), which is also essential for intercellular communication, signal transduction, proliferation and apoptosis (Cal et al., 2000). Oocytes form tight junctions with cumulus cells (CCs) in cumulus-oocyte complexes (COCs), allowing the flow of stimulatory factors required for oocyte maturation (Kempisty et al., 2014), and the oviduct has a similar role as gametes and embryos. Interestingly, a few DE circRNAs potentially regulating the viability and differentiation of sperm were enriched in biological processes, such as spermatogenesis and the exchange of chromosomal proteins, spermatid differentiation, and sperm motility. Before the sperm flow in the oviduct in the opposite direction and eventually combine with the egg, the oviduct activates sperm function to ensure that the egg can be fertilized, and most sperm adhere to the OEC before ovulation and are capacitated when ovulation occurs (Coy et al., 2012). Meanwhile, reproduction-related pathways were also enriched, including GnRH, insulin, progesterone-mediated oocyte maturation, and oocyte meiosis. Zhang et al. emphasized that the different levels of GnRH were among the reasons for the different number of lambs (Zhang et al., 2019). Insulin signaling pathway are an important KEGG terms, which has been suggested to participate in the regulation of reproduction (Burcelin et al., 2003). Oocyte meiosis is a crucial process involved in oocyte migration and fertilization, which are the primary activities of the oviduct, and related to the production and development of oocytes (Luvoni et al., 2003; Jang et al., 2022).

In this study, we found that DE circRNAs associated with reproduction were specifically enriched in different comparison groups. In the follicular phase (FL vs. FH), the host genes *SMAD1*, *MAPK8*, *IQCG*, *UBE2J1*, and *ATP9B* of chi_circ_0021996, chi_circ_0029140, chi_circ_0001504, chi_circ_0012952, and chi_circ_0027408 were involved in reproductive processes. *SMAD1* belongs to a family of receptor-activated proteins that mediate TGF-β superfamily ligand signalling, including TGF–beta and BMPs. In terms of embryonic haematopoiesis, *SMAD1* can act as a receptor for *SMADs* to regulate multiple steps of haematopoiesis, and *SMAD1*-depleted embryos will produce higher numbers of primitive erythrocytes, but the inability to produce mature embryonic macrophages will ultimately lead to failure of haematopoiesis progenitor cell production (McReynolds et al., 2007). *MAPK8* has been proven to be involved in germ cell apoptosis and redistribution of *BCL2-modifying factor* (*BMF*) (Show et al., 2008). In mice, *UBE2J1* deficiency significantly reduces sperm viability, which may have implications for fertilization and is responsible for a large kidding number (Koenig et al., 2014). In the luteal phase (LL vs. LH), the host genes of chi_circ_0000475, chi_circ_0013463, chi_circ_0000659, and chi_circ_0010484 were *RYK*, *ESR1*, *EVA1C,* and *SMAD5*, respectively. *RTK* is a member of the WNT signalling pathway receptor, and WNT has multiple biological functions and is highly correlated with female fertility (Habara et al., 2021). The *estrogen receptor alpha gene* (*ESR1*) is involved in hormonal regulation, whereas luteal phase OECs are predominantly secretory cells and are involved in estrogen secretion (Abe, 1996), and estrogen is an important regulator of reproduction (Oh et al., 2017). Furthermore, *ESR1* is an important transcriptional regulator in the mammalian oviduct and identified it as an important potential future regulator of fertility (Cerny et al., 2016). To better understand the molecular mechanisms of the goat prolificacy trait, understanding the function of DE circRNAs in the follicular-luteal phase transition is important. In the comparison group of the follicular–luteal phase transition (LH vs. FH and LL vs. FL group), chi_circ_0006485, chi_circ_002772, chi_circ_0020422, chi_circ_0022766, chi_circ_0001866, chi_circ_0027916, chi_circ_0030655, chi_circ_0025501, chi_circ_0013210, and chi_circ_0023024 were expressed, and their host genes *CUL1*, *ADCY9*, *PGR*, *GYS1*, *JAM2*, *FGFR2*, *MAPK1*, *MAPK3*, *MAP3K5*, and *FN1* were involved in the regulation of reproduction. *Cullin1* (*CUL1*) is a scaffold protein in cullin-based ubiquitin ligase, which plays important role in early embryonic development and in promoting the invasion of trophoblast into the maternal-fetal interface (Zhang et al., 2013). In mice, *CUL1* deficiency causes early embryonic lethality and dysregulates cell cycle regulatory protein (cyclin E) (Wang et al., 1999), which has a direct impact on fertility. Furthermore, endothelial cells expressing *adenylate cyclase 9* (*ADCY9*) were found to regulate endothelial cell signalling (Rautureau et al., 2022), and signalling is critical for gamete/embryo-maternal communication, which determines the success of early reproductive events (Alminana et al., 2018). Muscle-specific *GYS1* deficiency in adult mice results in poor glucose tolerance and reduced muscle glucose uptake due to insulin resistance as well as reduced exercise capacity and endurance, which is detrimental to cellular transport (Xirouchaki et al., 2016). The combined results of this study and those reported previously suggest that reproductive traits and embryonic development are a complex process that includes many oviductal events, where circRNAs distributed in the two estrous phases of high- and low-fecundity goats are mainly involved in embryonic development, hormonal regulation, reproductive events, and other biological processes.

It is well established that some circRNAs usually act as miRNA sponges, regulating the expression of target genes and affecting biological processes (Piwecka et al., 2017; Patop et al., 2019). To date, studies involving high-throughput sequencing in the oviduct have been limited, and few studies have included competitive endogenous RNAs. In this study, the circRNA-binding miRNAs were predicted as candidates using miRanda software. A total of 50 miRNAs might be sponged by 59 circRNAs, including 14 known miRNAs (Figure 5B). Notably, the high expression levels of miR-15a-5p inhibit granulosa cell proliferation by regulating the PI3K–AKT–mTOR signal pathway, promote apoptosis through *BCL2* and *BAD,* and are involved in the regulation of embryo quality (Zhang et al., 2017; Abu-Halima et al., 2020). MiR-181b-5p, an extraembryonic source of miRNA, might regulate embryo culture *in vitro* (Sanchez-Ribas et al., 2019). MiR-23b-5p plays a role in the regulation of brown adipogenesis and thermogenesis (You et al., 2020). MiR-204-3p may play a protective role in high-glucose-induced proapoptosis and dysfunction through the downregulation of Bdkrb2 (Han et al., 2019). Therefore, a ceRNA (circRNA–miRNA–mRNA) regulatory network were constructed based on embryonic development and reproduction-related signalling pathways. Interestingly, the embryonic development-related genes *SMAD1*, *TGFBR3*, *REV1*, and *BMP2K* were targeted by miR-145-5p, therefore, we focused on the chi_circ_0001504–chi-miR-145-5p–*SMAD1* axis (Figure 6C). Furthermore, we found that circIQCG is mainly localized in the cell and cytoplasm, and its host gene *IQCG* may control haematopoitetic stem cell (HSC) proliferation by interacting with calmodulin, and the final haematopoitetic function of *IQCG*-deficient embryos is severely impaired (Chen et al., 2012). Emerging evidence suggests that miR-145-5p can target *CD40* to regulate inflammatory responses and cardiomyocyte apoptosis and can inhibit goat myogenic cell differentiation by targeting *USP13* (Yuan et al., 2017; Zhang et al., 2022b). Hence, we hypothesize that circIQCG may acts as an mi145-5P sponge to promote embryonic development. Next, we demonstrate that the expression levels of chi_circ_0001504/*SMAD1* and miR-145-5p were opposite, suggesting that chi_circ_0001504 may act as miR-145-5p sponge to promote embryonic development by regulating the expression of *SMAD1*. Our findings provide data for further investigation of the mechanisms by which circRNAs regulate oviduct function and the interaction of embryonic development in goats. In conclusion, these DE circRNAs identified may regulate oviductal function by regulating miRNAs to accommodate the effect of different oestrus stages of the oviduct on kidding number; however, these regulatory mechanisms, however, need to be further validated at a later stage.

## Conclusion

In this study, the circRNA expression profiles of low- and high-fecundity Yunshang black goats were established, and numerous DE circRNAs, corresponding to varying amounts in oviducts with different estrous cycles, were annotated. The host genes involved in the reproduction were enriched in the various biological processes and pathways, such as gamete generation, embryonic cleavage, sperm motility, cell migration, focal adhesion, progesterone-mediated oocyte maturation, hormone secretion-related signalling pathways, oocyte meiosis, and PI3K-Akt, which significantly impact on gamete production, fertilization, and embryo development. In addition, the target miRNA sites in circRNAs for miR-15a-5p, miR-181b-5p, miR-23b-5p, miR-204-3p, and miR-145-5p were revealed through ceRNA network analysis. Furthermore, the present study identifies that circIQCG appears to be a probable biomarker for embryo development by affecting *SMAD1* expression by sponging miR-145-5p, thereby promoting the TGF-β pathway activation.

## Data Availability

The datasets presented in this study can be found in online repositories. The name of the repository and accession number can be found below: SRA, NCBI BioProject PRJNA854769.

## References

[B1] Abdelmohsen K. Panda A. C. Munk R. Grammatikakis I. Dudekula D. B. De S. (2017). Identification of HuR target circular RNAs uncovers suppression of PABPN1 translation by CircPABPN1. RNA Biol. 14 (3), 361–369. 10.1080/15476286.2017.1279788 28080204 PMC5367248

[B2] Abe H. Oikawa T. (1992). Examination by scanning electron microscopy of oviductal epithelium of the prolific Chinese meishan pig at follicular and luteal phases. Anat. Rec. 233 (3), 399–408. 10.1002/ar.1092330307 1609972

[B3] Abe H. Oikawa T. (1993). Observations by scanning electron microscopy of oviductal epithelial cells from cows at follicular and luteal phases. Anat. Rec. 235 (3), 399–410. 10.1002/ar.1092350309 8430910

[B4] Abe H. (1996). The mammalian oviductal epithelium: Regional variations in cytological and functional aspects of the oviductal secretory cells. Histol. Histopathol. 11 (3), 743–768. 8839764

[B5] Abu-Halima M. Khaizaran Z. A. Ayesh B. M. Fischer U. Khaizaran S. A. Al-Battah F. (2020). MicroRNAs in combined spent culture media and sperm are associated with embryo quality and pregnancy outcome. Fertil. Steril. 113 (5), 970–980.e972. 10.1016/j.fertnstert.2019.12.028 32222254

[B6] Alminana C. Tsikis G. Labas V. Uzbekov R. da Silveira J. C. Bauersachs S. (2018). Deciphering the oviductal extracellular vesicles content across the estrous cycle: Implications for the gametes-oviduct interactions and the environment of the potential embryo. BMC Genomics 19 (1), 622. 10.1186/s12864-018-4982-5 30134841 PMC6103977

[B7] Baumann K. (2020). CircRNAs in lifespan. Nat. Rev. Mol. Cell Biol. 21 (8), 420. 10.1038/s41580-020-0269-1 32616909

[B8] Budna-Tukan J. Swiatly-Blaszkiewicz A. Celichowski P. Kaluzna S. Konwerska A. Sujka-Kordowska P. (2019). Biological adhesion" is a significantly regulated molecular process during long-term primary *in vitro* culture of oviductal epithelial cells (oecs): A transcriptomic and proteomic study. Int. J. Mol. Sci. 20 (14), E3387. 10.3390/ijms20143387 31295879 PMC6678391

[B9] Burcelin R. Thorens B. Glauser M. Gaillard R. C. Pralong F. P. (2003). Gonadotropin-releasing hormone secretion from hypothalamic neurons: Stimulation by insulin and potentiation by leptin. Endocrinology 144 (10), 4484–4491. 10.1210/en.2003-0457 12960084

[B10] Cal S. Freije J. M. Lopez J. M. Takada Y. Lopez-Otin C. (2000). ADAM 23/MDC3, a human disintegrin that promotes cell adhesion via interaction with the Alphavbeta3 integrin through an RGD-independent mechanism. Mol. Biol. Cell 11 (4), 1457–1469. 10.1091/mbc.11.4.1457 10749942 PMC14859

[B11] Cerny K. L. Ribeiro R. A. Jeoung M. Ko C. Bridges P. J. (2016). Estrogen receptor alpha (ESR1)-Dependent regulation of the mouse oviductal transcriptome. PLoS One 11 (1), e0147685. 10.1371/journal.pone.0147685 26808832 PMC4725743

[B12] Chang H. J. Shin H. S. Kim T. H. Yoo J. Y. Teasley H. E. Zhao J. J. (2018). Pik3ca is required for mouse uterine gland development and pregnancy. PLoS One 13 (1), e0191433. 10.1371/journal.pone.0191433 29346447 PMC5773209

[B13] Chen L. T. Liang W. X. Chen S. Chen Z. Chen S. J. (2012). A calmodulin interacting protein iqcg is required for definitive hematopoiesis in zebrafish. Blood 120 (21), 764. 10.1182/blood.V120.21.764.764

[B14] Chen Y. H. Yu M. Podd A. Wen R. Chrzanowska-Wodnicka M. White G. C. (2008). A critical role of Rap1b in B-cell trafficking and marginal zone B-cell development. Blood 111 (9), 4627–4636. 10.1182/blood-2007-12-128140 18319399 PMC2343596

[B15] Coy P. Garcia-Vazquez F. A. Visconti P. E. Aviles M. (2012). Roles of the oviduct in mammalian fertilization. Reproduction 144 (6), 649–660. 10.1530/rep-12-0279 23028122 PMC4022750

[B16] Dang Y. J. Yan L. Y. Hu B. Q. Fan X. Y. Ren Y. X. Li R. (2016). Tracing the expression of circular RNAs in human pre-implantation embryos. Genome Biol. 17 (1), 130. 10.1186/s13059-016-0991-3 27315811 PMC4911693

[B17] Gao Y. Zhang J. Y. Zhao F. Q. (2018). Circular RNA identification based on multiple seed matching. Brief. Bioinform. 19 (5), 803–810. 10.1093/bib/bbx014 28334140

[B18] Habara O. Logan C. Y. Kanai-Azuma M. Nusse R. Takase H. M. (2021). WNT signaling in pre-granulosa cells is required for ovarian folliculogenesis and female fertility. Development 148 (9), dev198846. 10.1242/dev.198846 33914868 PMC8126407

[B19] Han X. Li Q. B. Wang C. Y. Li Y. Y. (2019). MicroRNA-204-3p attenuates high glucose-induced MPC5 podocytes apoptosis by targeting braykinin B2 receptor. Exp. Clin. Endocrinol. Diabetes 127 (6), 387–395. 10.1055/a-0630-0173 29940664

[B20] Hess A. P. Talbi S. Hamilton A. E. Baston-Buest D. M. Nyegaard M. Irwin J. C. (2013). The human oviduct transcriptome reveals an anti-inflammatory, anti-angiogenic, secretory and matrix-stable environment during embryo transit. Reprod. Biomed. Online 27 (4), 423–435. 10.1016/j.rbmo.2013.06.013 23953067 PMC3950339

[B21] Hunter R. H. (2003). Reflections upon sperm-endosalpingeal and sperm-zona pellucida interactions *in vivo* and *in vitro* . Reprod. Domest. Anim. 38 (2), 147–154. 10.1046/j.1439-0531.2003.00402.x 12654026

[B22] Jang M. J. Lim C. Lim B. Kim J. M. (2022). Integrated multiple transcriptomes in oviductal tissue across the porcine estrous cycle reveal functional roles in oocyte maturation and transport. J. Anim. Sci. 100 (2), skab364. 10.1093/jas/skab364 34918099 PMC8846367

[B23] Jeck W. R. Sharpless N. E. (2014). Detecting and characterizing circular RNAs. Nat. Biotechnol. 32 (5), 453–461. 10.1038/nbt.2890 24811520 PMC4121655

[B24] John B. Enright A. J. Aravin A. Tuschl T. Sander C. Marks D. S. (2005). Human MicroRNA targets. PLoS Biol. 3 (7), e363. 10.1371/journal.pbio.0020363 15502875 PMC521178

[B25] Kempisty B. Ziolkowska A. Ciesiolka S. Piotrowska H. Antosik P. Bukowska D. (2014). Study on connexin gene and protein expression and cellular distribution in relation to real-time proliferation of porcine granulosa cells. J. Biol. Regul. Homeost. Agents 28 (4), 625–635. 25620173

[B26] Kim D. Langmead B. Salzberg S. L. (2015). Hisat: A fast spliced aligner with low memory requirements. Nat. Methods 12 (4), 357–360. 10.1038/nmeth.3317 25751142 PMC4655817

[B27] Koenig P. A. Nicholls P. K. Schmidt F. I. Hagiwara M. Maruyama T. Frydman G. H. (2014). The E2 ubiquitin-conjugating enzyme UBE2J1 is required for spermiogenesis in mice. J. Biol. Chem. 289 (50), 34490–34502. 10.1074/jbc.M114.604132 25320092 PMC4263858

[B28] Li B. J. He Y. Wu W. J. Tan X. F. Wang Z. C. H. Irwin D. M. (2022). Circular RNA profiling identifies novel circPPARA that promotes intramuscular fat deposition in pigs. J. Agric. Food Chem. 70 (13), 4123–4137. 10.1021/acs.jafc.1c07358 35324170

[B29] Li H. Durbin R. (2009). Fast and accurate short read alignment with burrows-wheeler transform. Bioinformatics 25 (14), 1754–1760. 10.1093/bioinformatics/btp324 19451168 PMC2705234

[B30] Li T. T. Luo R. R. Wang X. Wang H. H. Zhao X. X. Guo Y. X. (2021). Unraveling stage-dependent expression patterns of circular RNAs and their related ceRNA modulation in ovine postnatal testis development. Front. Cell Dev. Biol. 9, 627439. 10.3389/fcell.2021.627439 33816472 PMC8017185

[B31] Li Z. Y. Huang C. Bao C. Chen L. Lin M. Wang X. L. (2015). Exon-intron circular RNAs regulate transcription in the nucleus. Nat. Struct. Mol. Biol. 22 (3), 256–264. 10.1038/nsmb.2959 25664725

[B32] Liang D. Wilusz J. E. (2014). Short intronic repeat sequences facilitate circular RNA production. Genes Dev. 28 (20), 2233–2247. 10.1101/gad.251926.114 25281217 PMC4201285

[B33] Lima L. G. D. Souza N. O. B. D. Rios R. R. Melo B. A. D. Santos L. T. A. D. Silva K. D. M. (2020). Advances in molecular genetic techniques applied to selection for litter size in goats (*Capra hircus*): A review. J. Appl. Animal Res. 48 (1), 38–44. 10.1080/09712119.2020.1717497

[B34] Liu C. X. Li X. Nan F. Jiang S. Gao X. Guo S. K. (2019). Structure and degradation of circular RNAs regulate PKR activation in innate immunity. Cell 177 (4), 865–880.e821. 10.1016/j.cell.2019.03.046 31031002

[B35] Liu M. Wang Y. Q. Lu H. F. Wang H. Shi X. M. Shao X. (2018). miR-518b enhances human trophoblast cell proliferation through targeting Rap1b and activating ras-MAPK signal. Front. Endocrinol. 9, 100. 10.3389/fendo.2018.00100 PMC5862798 29599749

[B36] Liu R. L. Liu X. X. Bai X. J. Xiao C. Z. Dong Y. J. (2020). Identification and characterization of circRNA in longissimus dorsi of different breeds of cattle. Front. Genet. 11, 565085. 10.3389/fgene.2020.565085 33324445 PMC7726199

[B37] Liu X. R. Zhang L. Cui J. Z. Yang L. C. Han J. C. Che S. C. (2021). circRNA landscape of non-pregnant endometrium during the estrus cycle in dairy goats. J. Integr. Agric. 20 (5), 1346–1358. 10.1016/S2095-3119(20)63464-5

[B38] Liu X. R. X. R. Zhang L. Liu Y. X. Cui J. Z. Che S. C. An X. P. (2018). Circ-8073 regulates CEP55 by sponging miR-449a to promote caprine endometrial epithelial cells proliferation via the PI3K/AKT/mTOR pathway. Biochim. Biophys. Acta. Mol. Cell Res. 1865 (8), 1130–1147. 10.1016/j.bbamcr.2018.05.011 29800603

[B39] Liu Y. F. Wang P. Zhou Z. Y. He X. Y. Tao L. Jiang Y. T. (2021). Expression profile Analysis to identify circular RNA expression signatures in the prolificacy trait of Yunshang black goat pituitary in the estrus cycle. Front. Genet. 12, 801357. 10.3389/fgene.2021.801357 35140742 PMC8820483

[B40] Liu Y. F. Zhou Z. Y. He X. Y. Jiang Y. T. Ouyang Y. N. Hong Q. H. (2022). Differentially expressed circular RNA profile signatures identified in prolificacy trait of Yunshang black goat ovary at estrus cycle. Front. Physiol. 13, 820459. 10.3389/fphys.2022.820459 35492611 PMC9049588

[B41] Luo J. Wang W. Sun S. (2019). Research advances in reproduction for dairy goats. Asian-Australas. J. Anim. Sci. 32 (8), 1284–1295. 10.5713/ajas.19.0486 31357269 PMC6668861

[B42] Luvoni G. C. Chigioni S. Allievi E. Macis D. (2003). Meiosis resumption of canine oocytes cultured in the isolated oviduct. Reprod. Domest. Anim. 38 (5), 410–414. 10.1046/j.1439-0531.2003.00457.x 12950695

[B43] Lv X. Y. Chen W. H. Sun W. Hussain Z. Chen L. Wang S. H. (2020). Expression profile Analysis to identify circular RNA expression signatures in hair follicle of hu sheep lambskin. Genomics 112 (6), 4454–4462. 10.1016/j.ygeno.2020.07.046 32768426

[B44] Lyons R. A. Saridogan E. Djahanbakhch O. (2006). The reproductive significance of human fallopian tube cilia. Hum. Reprod. Update 12 (4), 363–372. 10.1093/humupd/dml012 16565155

[B45] Ma L. Zhang M. Cao F. J. Han J. C. Han P. Wu Y. T. (2022). Effect of MiR-100-5p on proliferation and apoptosis of goat endometrial stromal cell *in vitro* and embryo implantation *in vivo* . J. Cell. Mol. Med. 26 (9), 2543–2556. 10.1111/jcmm.17226 35411593 PMC9077292

[B46] Mao X. Z. Cai T. Olyarchuk J. G. Wei L. P. (2005). Automated genome annotation and pathway identification using the KEGG orthology (KO) as A controlled vocabulary. Bioinformatics 21 (19), 3787–3793. 10.1093/bioinformatics/bti430 15817693

[B47] McReynolds L. J. Gupta S. Figueroa M. E. Mullins M. C. Evans T. (2007). Smad1 and Smad5 differentially regulate embryonic hematopoiesis. Blood 110 (12), 3881–3890. 10.1182/blood-2007-04-085753 17761518 PMC2200801

[B48] Memczak S. Jens M. Elefsinioti A. Torti F. Krueger J. Rybak A. (2013). Circular RNAs are a large class of animal RNAs with regulatory potency. Nature 495 (7441), 333–338. 10.1038/nature11928 23446348

[B49] Notter D. R. (2012). Genetic improvement of reproductive efficiency of sheep and goats. Anim. Reprod. Sci. 130 (3-4), 147–151. 10.1016/j.anireprosci.2012.01.008 22325926

[B50] Oh Y. S. Koh I. K. Choi B. Gye M. C. (2017). ESR1 inhibits hCG-induced steroidogenesis and proliferation of progenitor leydig cells in mice. Sci. Rep. 7, 43459. 10.1038/srep43459 28266530 PMC5339920

[B51] Patop I. L. Wust S. Kadener S. (2019). Past, present, and future of circRNAs. Embo J. 38 (16), e100836. 10.15252/embj.2018100836 31343080 PMC6694216

[B52] Piwecka M. Glazar P. Hernandez-Miranda L. R. Memczak S. Wolf S. A. Rybak-Wolf A. (2017). Loss of a mammalian circular RNA locus causes miRNA deregulation and affects brain function. Science 357 (6357), eaam8526. 10.1126/science.aam8526 28798046

[B53] Pramod R. Sharma S. Singhi A. Pan S. Mitra A. (2013). Differential ovarian morphometry and follicular expression of BMP15, GDF9 and BMPR1B influence the prolificacy in goat. Reprod. Dom. Anim. 48 (5), 803–809. 10.1111/rda.12165 23581245

[B54] Rautureau Y. Berlatie M. Rivas D. Uy K. Blanchette A. Miquel G. (2022). Adenylate cyclase type 9 antagonizes cAMP accumulation and regulates endothelial signalling involved in atheroprotection. Cardiovasc. Res., cvac085. 10.1093/cvr/cvac085 35576489

[B55] Salzman J. Chen R. E. Olsen M. N. Wang P. L. Brown P. O. (2013). Cell-type specific features of circular RNA expression. PLoS Genet. 9 (9), e1003777. 10.1371/journal.pgen.1003777 24039610 PMC3764148

[B56] Sanchez-Ribas I. Diaz-Gimeno P. Quinonero A. Ojeda M. Larreategui Z. Ballesteros A. (2019). NGS analysis of human embryo culture media reveals miRNAs of extra embryonic origin. Reprod. Sci. 26 (2), 214–222. 10.1177/1933719118766252 29587610

[B57] Sharma R. K. (2000). Follicular atresia in goat: A review. Ind. J. Anim. Sci. 70 (10), 1035–1046. 10.1016/S0739-7240(00)00073-4

[B58] Show M. D. Hill C. M. Anway M. D. Wright W. W. Zirkin B. R. (2008). Phosphorylation of mitogen-activated protein kinase 8 (MAPK8) is associated with germ cell apoptosis and redistribution of the bcl2-modifying factor (BMF). J. Androl. 29 (3), 338–344. 10.2164/jandrol.107.003558 18222916

[B59] Silva J. R. V. van den Hurk R. van Tol H. T. A. Roelen B. A. J. Figueiredo J. R. (2005). Expression of growth differentiation factor 9 (GDF9), bone morphogenetic protein 15 (BMP15), and BMP receptors in the ovaries of goats. Mol. Reprod. Dev. 70 (1), 11–19. 10.1002/mrd.20127 15515056

[B60] Tao H. Xiong Q. Zhang F. Zhang N. Liu Y. Suo X. J. (2017). Circular RNA profiling reveals chi_circ_0008219 function as micro RNA sponges in pre-ovulatory ovarian follicles of goats (*Capra hircus*). Genomics 110 (4), 257–266. 10.1016/j.ygeno.2017.10.005 29107014

[B61] Vo J. N. Cieslik M. Zhang Y. Shukla S. Xiao L. Zhang Y. (2019). The landscape of circular RNA in cancer. Cell 176 (4), 869–881.e813. 10.1016/j.cell.2018.12.021 30735636 PMC6601354

[B62] Wang K. Liu X. F. Qi T. Hui Y. Q. Yan H. L. Qu L. (2021). Whole-genome sequencing to identify candidate genes for litter size and to uncover the variant function in goats (*Capra hircus*). Genomics 113 (1), 142–150. 10.1016/j.ygeno.2020.11.024 33276007

[B63] Wang Y. S. Penfold S. Tang X. J. Hattori N. Riley P. Harper J. W. (1999). Deletion of the Cul1 gene in mice causes arrest in early embryogenesis and accumulation of cyclin E. Curr. Biol. 9 (20), 1191–1194. 10.1016/s0960-9822(00)80024-x 10531039

[B64] Wilusz J. E. (2018). A 360° view of circular RNAs: From biogenesis to functions. Wiley Interdiscip. Rev. RNA 9 (4), e1478. 10.1002/wrna.1478 29655315 PMC6002912

[B65] Xirouchaki C. E. Mangiafico S. P. Bate K. Ruan Z. Huang A. M. Tedjosiswoyo B. W. (2016). Impaired glucose metabolism and exercise capacity with muscle-specific glycogen synthase 1 (gys1) deletion in adult mice. Mol. Metab. 5 (3), 221–232. 10.1016/j.molmet.2016.01.004 26977394 PMC4770268

[B66] You L. H. Wang Y. Gao Y. Wang X. Y. Cui X. W. Zhang Y. Y. (2020). The role of microRNA-23b-5p in regulating Brown adipogenesis and thermogenic program. Endocr. Connect. 9 (5), 457–470. 10.1530/ec-20-0124 32348962 PMC7274556

[B67] You X. T. Vlatkovic I. Babic A. Will T. Epstein I. Tushev G. (2015). Neural circular RNAs are derived from synaptic genes and regulated by development and plasticity. Nat. Neurosci. 18 (4), 603–610. 10.1038/nn.3975 25714049 PMC4376664

[B68] Yuan M. Zhang L. W. You F. Zhou J. Y. Ma Y. J. Yang F. F. (2017). MiR-145-5p regulates hypoxia-induced inflammatory response and apoptosis in cardiomyocytes by targeting CD40. Mol. Cell. Biochem. 431 (1-2), 123–131. 10.1007/s11010-017-2982-4 28281187

[B69] Zang J. K. Lu D. Xu A. D. (2020). The interaction of circRNAs and RNA binding proteins: An important part of circRNA maintenance and function. J. Neurosci. Res. 98 (1), 87–97. 10.1002/jnr.24356 30575990

[B70] Zhang K. Y. Zhong W. X. Li W. P. Chen Z. J. Zhang C. (2017). miR-15a-5p levels correlate with poor ovarian response in human follicular fluid. Reproduction 154 (4), 483–496. 10.1530/rep-17-0157 28729464

[B71] Zhang L. Liu X. R. Ma X. N. Liu Y. X. Che S. C. Cui J. Z. (2018). Testin was regulated by circRNA3175-miR182 and inhibited endometrial epithelial cell apoptosis in pre-receptive endometrium of dairy goats. J. Cell. Physiol. 233 (10), 6965–6974. 10.1002/jcp.26614 29693265

[B72] Zhang M. L. Zhao K. Xu X. P. Yang Y. B. Yan S. Wei P. (2018). A peptide encoded by circular form of LINC-PINT suppresses oncogenic transcriptional elongation in glioblastoma. Nat. Commun. 9 (1), 4475. 10.1038/s41467-018-06862-2 30367041 PMC6203777

[B73] Zhang Q. Chen Q. Lu X. Zhou Z. Zhang H. Lin H. Y. (2013). CUL1 promotes trophoblast cell invasion at the maternal-fetal interface. Cell Death Dis. 4 (2), e502. 10.1038/cddis.2013.1 23429288 PMC3734813

[B74] Zhang Z. B. Tang J. S. Di R. Liu Q. Y. Wang X. Y. Gan S. Q. (2019). Comparative transcriptomics reveal key sheep (ovis aries) hypothalamus lncRNAs that affect reproduction. Animals. 9, 152. 10.3390/ani9040152 PMC6523726 30965601

[B75] Zhang Z. Deng K. P. Kang Z. Q. Wang F. Fan Y. X. (2022b). MicroRNA profiling reveals miR-145-5p inhibits goat myoblast differentiation by targeting the coding domain sequence of USP13. Faseb J. 36 (7), e22370. 10.1096/fj.202200246R 35635726

[B76] Zhang Z. Fan Y. X. Deng K. P. Liang Y. X. Zhang G. M. Gao X. X. (2022c). Circular RNA circUSP13 sponges miR-29c to promote differentiation and inhibit apoptosis of goat myoblasts by targeting IGF1. Faseb J. 36 (1), e22097. 10.1096/fj.202101317R 34935184

[B77] Zhao Z. F. Zou X. Lu T. T. Deng M. Li Y. K. Guo Y. Q. (2020). Identification of mRNAs and lncRNAs involved in the regulation of follicle development in goat. Front. Genet. 11, 589076. 10.3389/fgene.2020.589076 33391342 PMC7773919

[B78] Zheng Q. P. Bao C. Y. Guo W. J. Li S. Y. Chen J. Chen B. (2016). Circular RNA profiling reveals an abundant circHIPK3 that regulates cell growth by sponging multiple miRNAs. Nat. Commun. 7, 11215. 10.1038/ncomms11215 27050392 PMC4823868

[B79] Zheng Y. Y. Hui T. Y. Yue C. Sun J. M. Guo D. Guo S. L. (2020). Comprehensive analysis of circRNAs from cashmere goat skin by Next generation RNA sequencing (RNA-seq). Sci. Rep. 10 (1), 516. 10.1038/s41598-019-57404-9 31949277 PMC6965140

[B80] Zou X. Lu T. T. Zhao Z. F. Liu G. B. Lian Z. Q. Guo Y. Q. (2020). Comprehensive analysis of mRNAs and miRNAs in the ovarian follicles of uniparous and multiple goats at estrus phase. BMC Genomics 21 (1), 267. 10.1186/s12864-020-6671-4 32228439 PMC7106838

