# Supplementary material for: Characterization of circular RNA profiles of oviduct reveal the potential mechanism in prolificacy trait of goat in the estrus cycle
Source: Front Physiol. 2022 Sep 15;13:990691. doi: 10.3389/fphys.2022.990691 (PMC9521424; doi:10.3389/fphys.2022.990691)
Supplement: Supplementary file 3 [file DataSheet1.PDF]

A

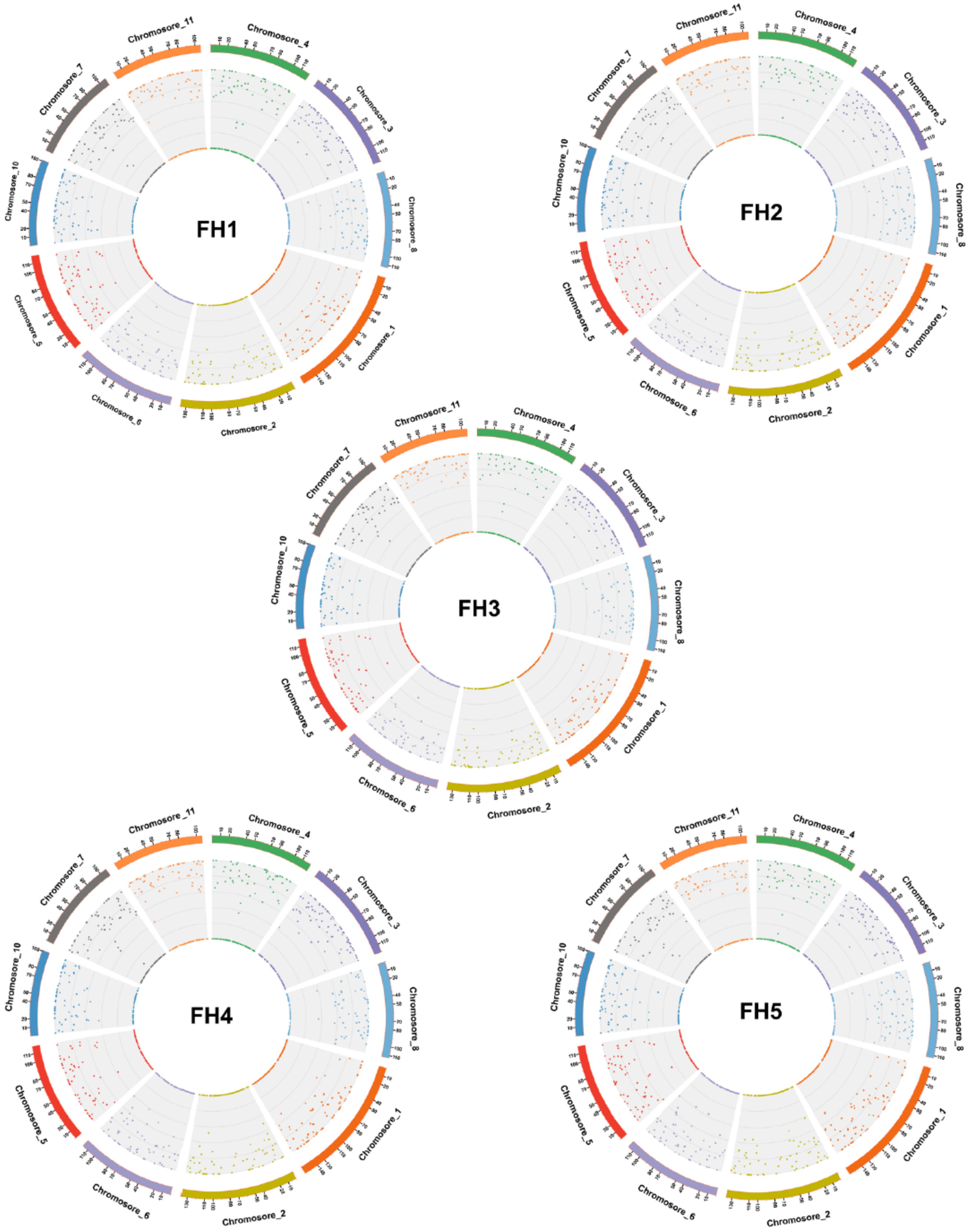
FH1
FH2
FH3
FH4
FH5
Chromosore_1
Chromosore_2
Chromosore_3
Chromosore_4
Chromosore_5
Chromosore_6
Chromosore_7
Chromosore_8
Chromosore_10
Chromosore_11

B

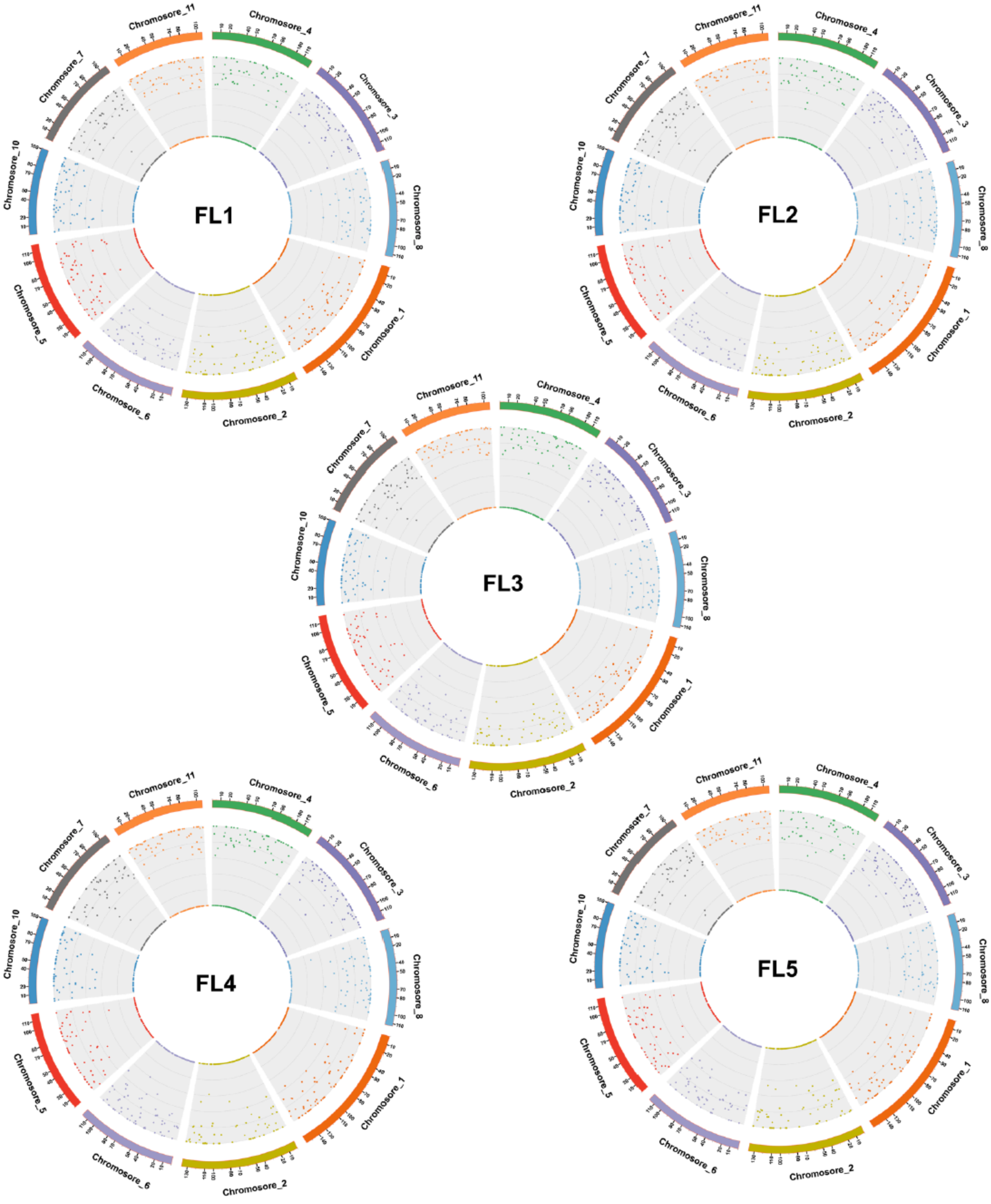
FL1
Chromosore_11
Chromosore_4
Chromosore_3
Chromosore_8
Chromosore_1
Chromosore_2
Chromosore_6
Chromosore_5
Chromosore_10
Chromosore_7
FL2
Chromosore_11
Chromosore_4
Chromosore_3
Chromosore_8
Chromosore_1
Chromosore_2
Chromosore_6
Chromosore_5
Chromosore_10
Chromosore_7
FL3
Chromosore_11
Chromosore_4
Chromosore_3
Chromosore_8
Chromosore_1
Chromosore_2
Chromosore_6
Chromosore_5
Chromosore_10
Chromosore_7
FL4
Chromosore_11
Chromosore_4
Chromosore_3
Chromosore_8
Chromosore_1
Chromosore_2
Chromosore_6
Chromosore_5
Chromosore_10
Chromosore_7
FL5
Chromosore_11
Chromosore_4
Chromosore_3
Chromosore_8
Chromosore_1
Chromosore_2
Chromosore_6
Chromosore_5
Chromosore_10
Chromosore_7

C

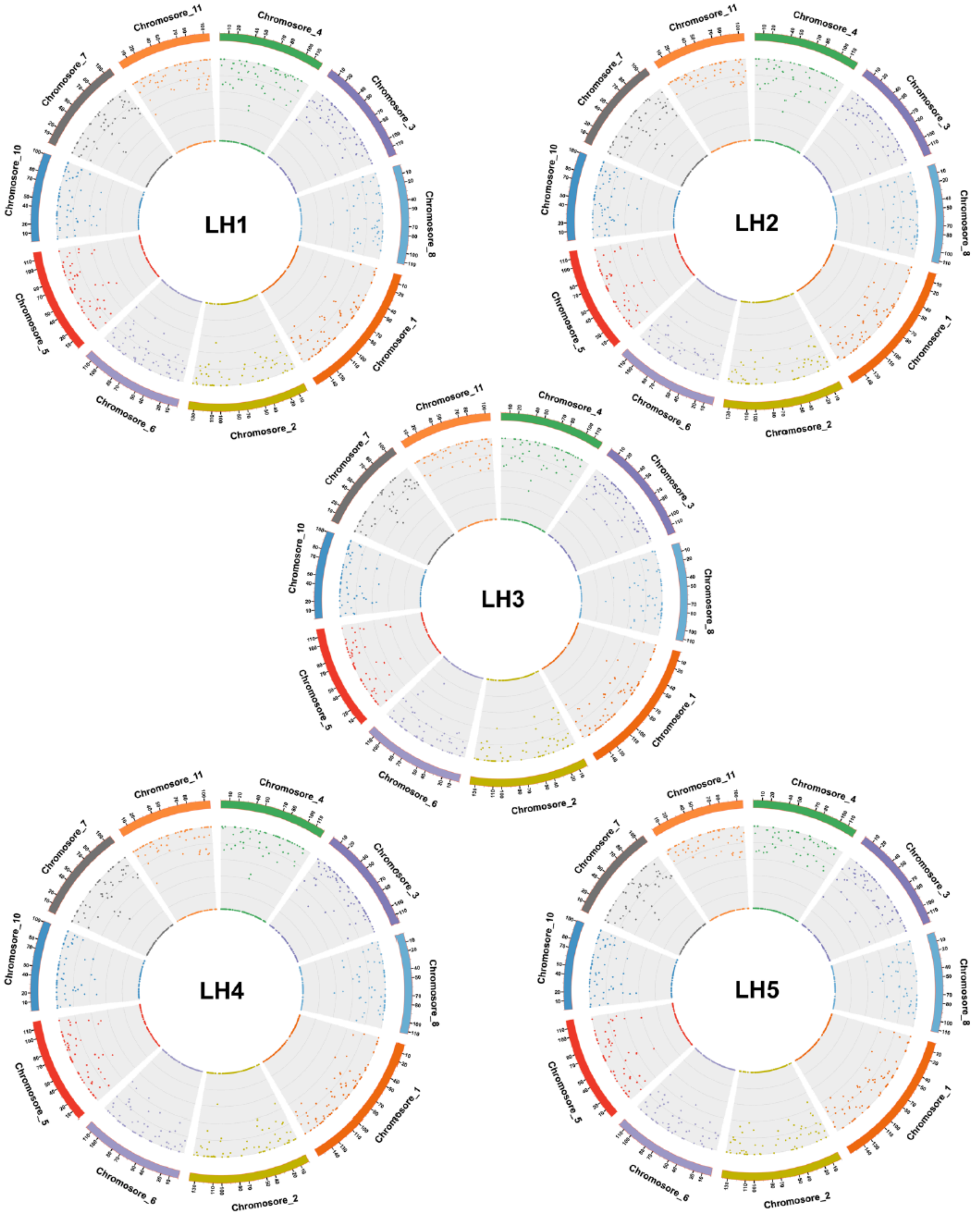
LH1
LH2
LH3
LH4
LH5
Chromosore_1
Chromosore_2
Chromosore_3
Chromosore_4
Chromosore_5
Chromosore_6
Chromosore_7
Chromosore_8
Chromosore_10
Chromosore_11

D

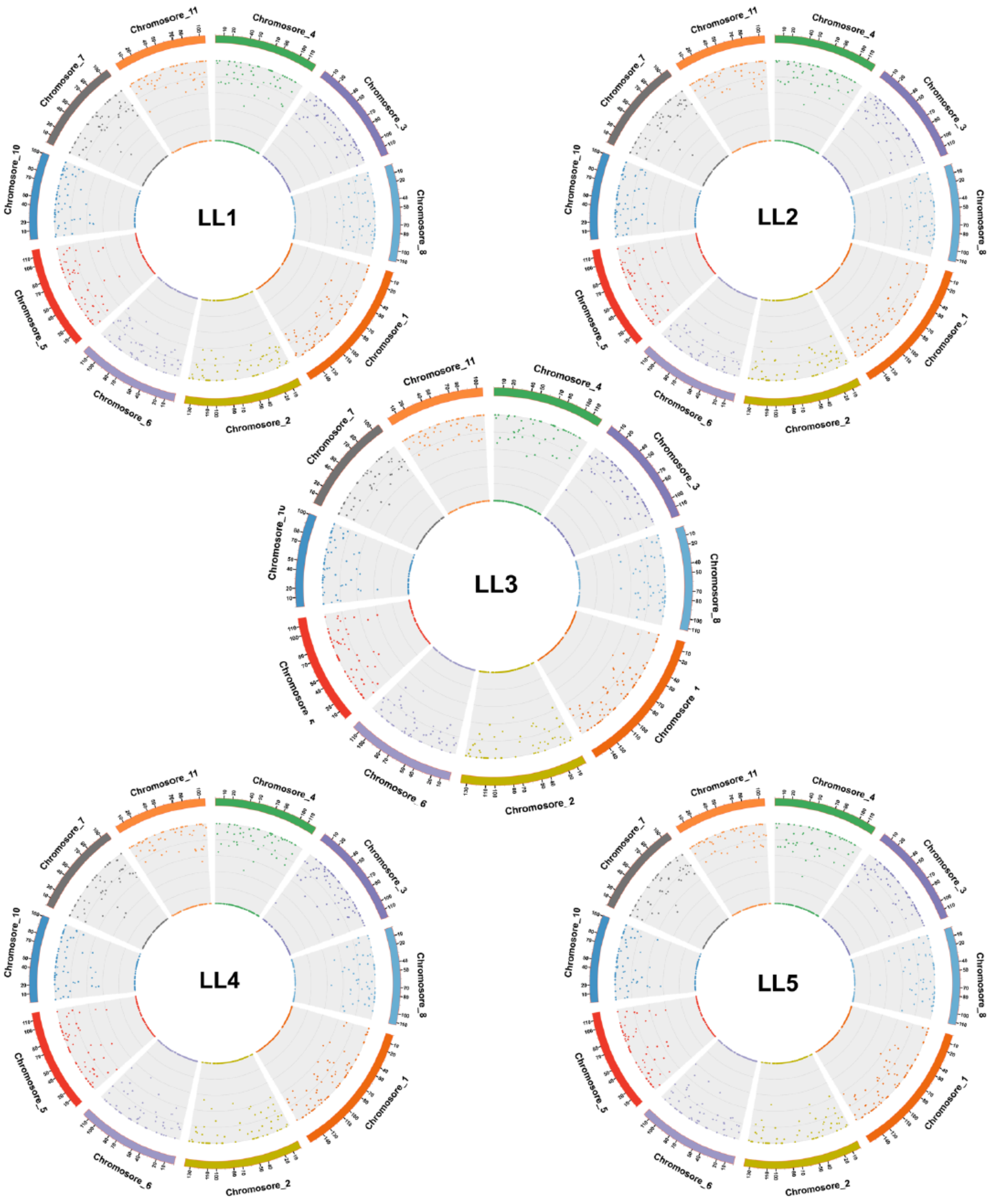

Figure S1. The distribution and expression of circRNAs on goat chromosomes in the four groups. (**A**) High-fecundity groups in the follicular phase (FH); (**B**) Low-fecundity groups in the follicular phase (FL); (**C**) High-fecundity groups in the luteal phase; (**D**) Low-fecundity groups in the luteal phase.

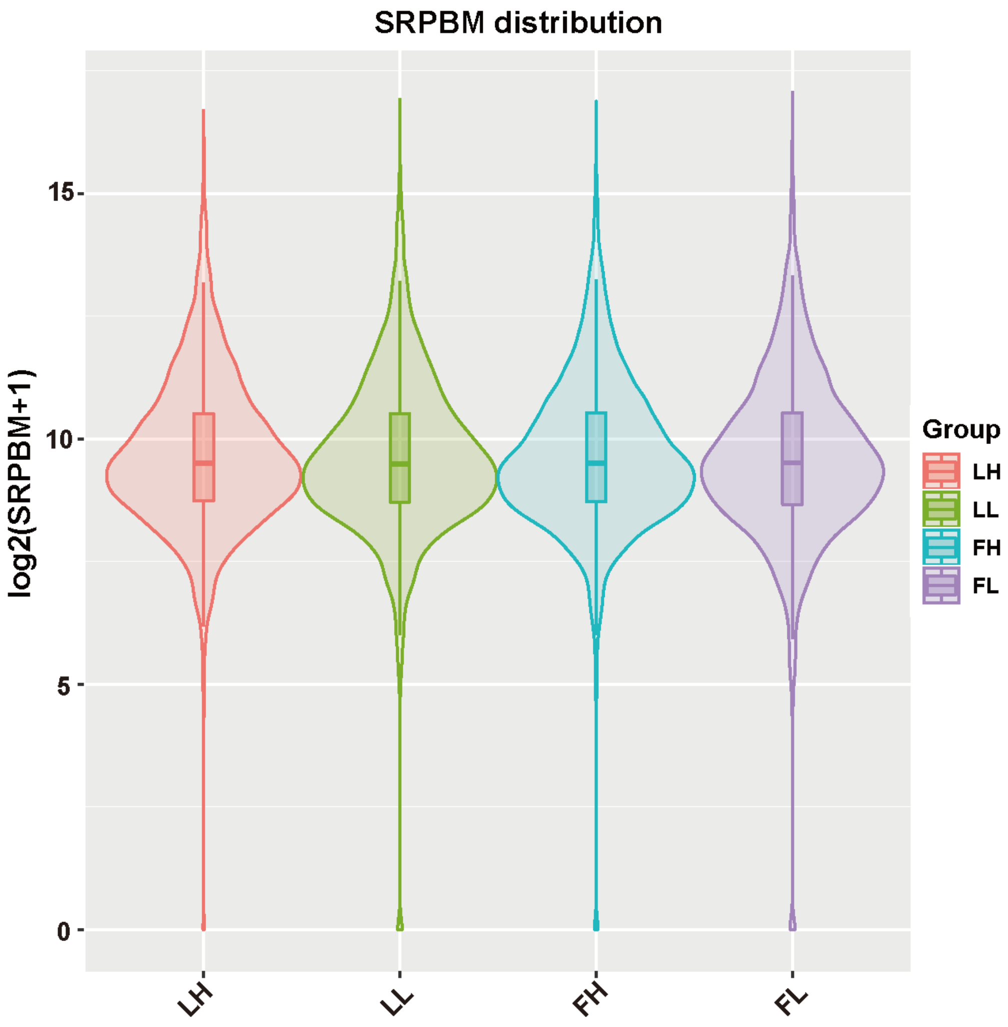

Figure S2. Violin plot of relative abundance of circRNAs in four oviduct tissue groups. Data are expressed as $\log_2$ (SRPBM+1).

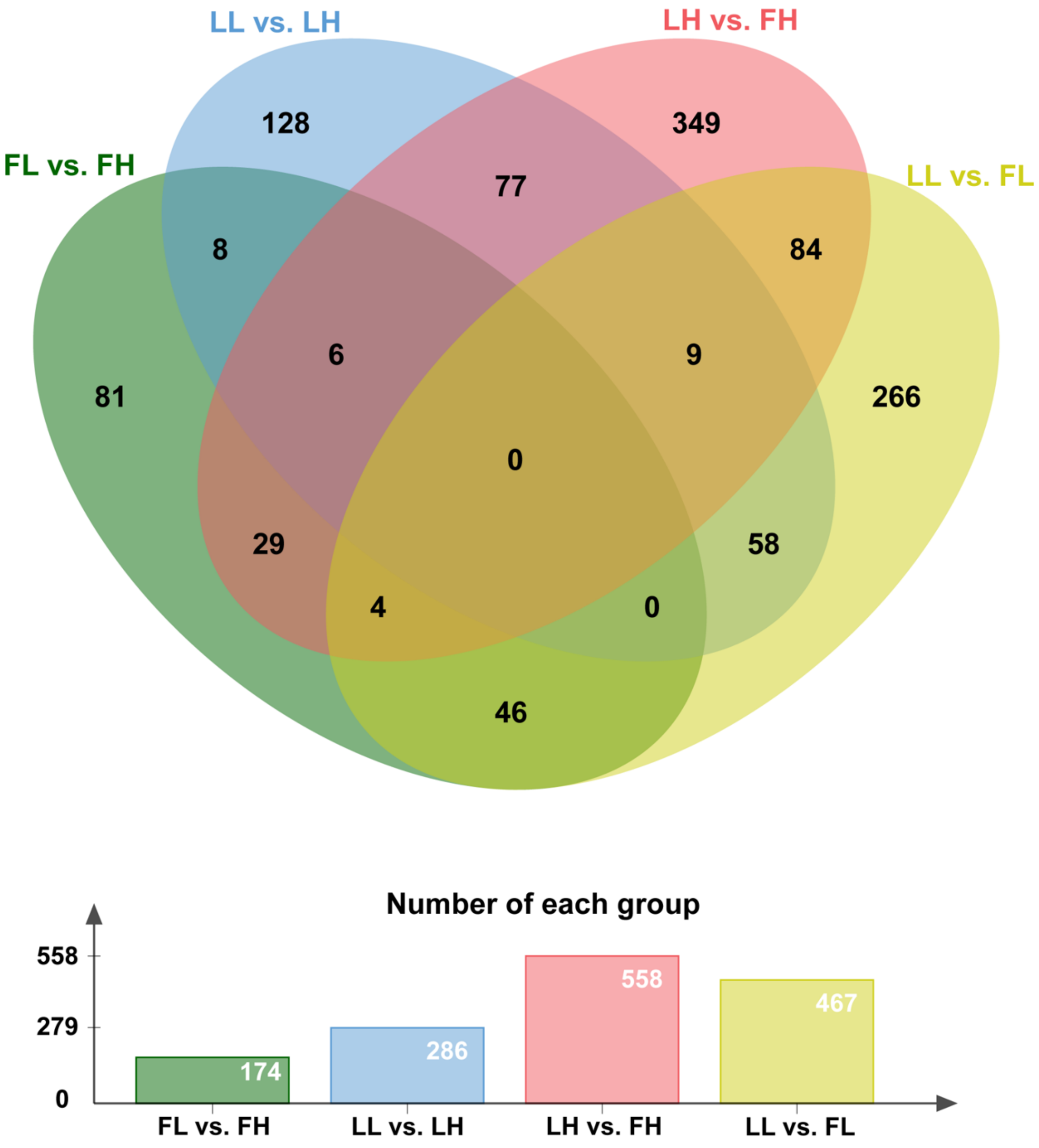

Figure S3. Venn diagram exhibiting overlapping of the circRNAs of LL vs. FL, FL vs. FH, LH vs. FH, LL vs. LH groups.

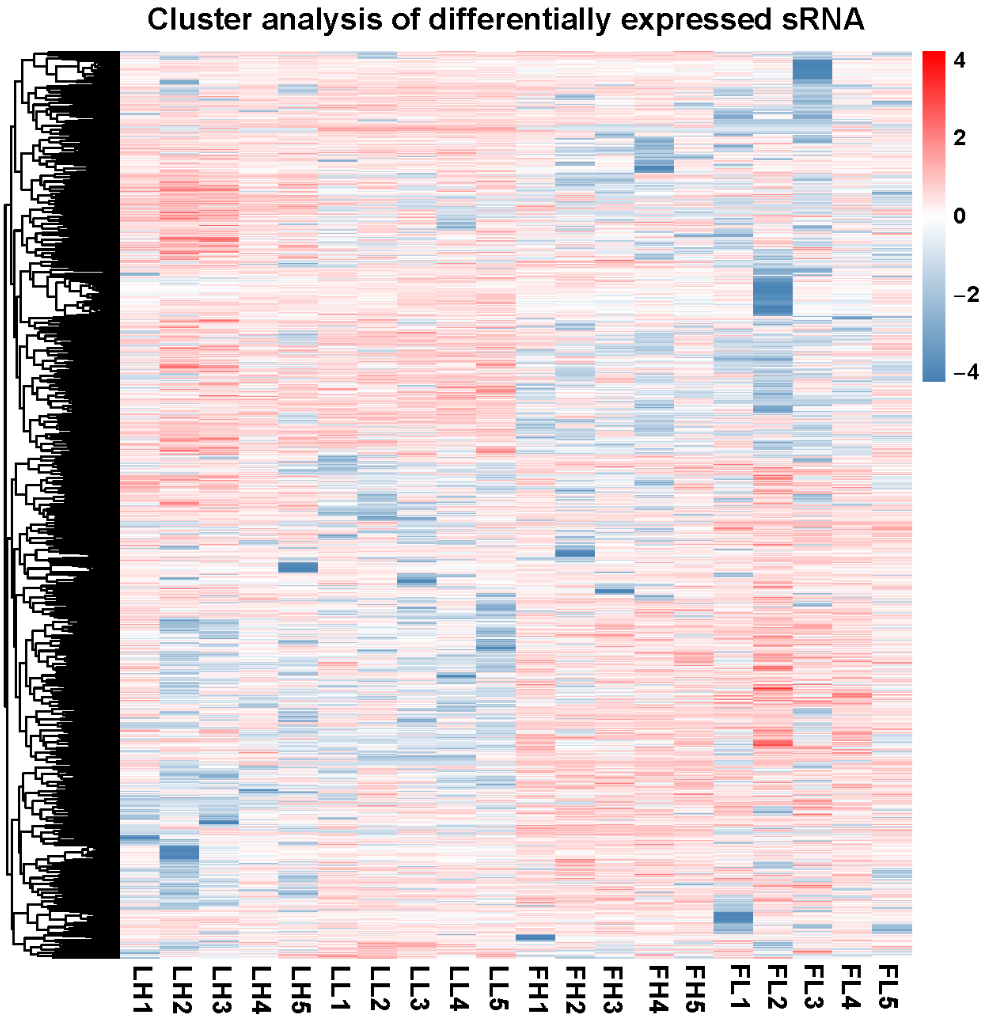

Figure S4. Heat map of circRNAs differentially expressed in oviduct tissues. The red and blue strips represented high and low expressions, respectively.

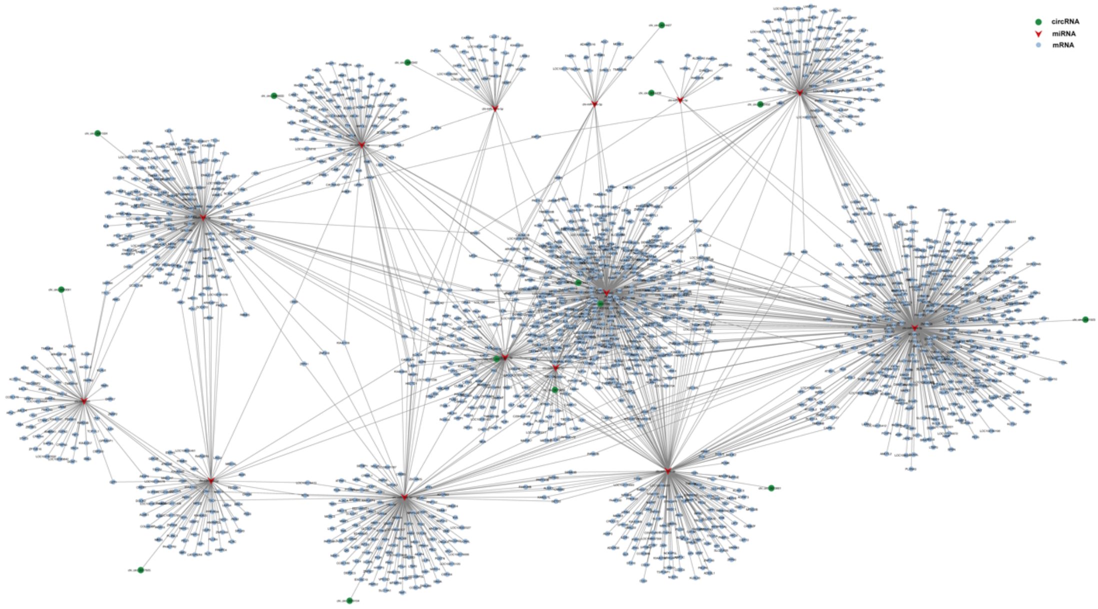

Figure S5. The circRNA–miRNA–mRNA interaction network.
